# One Cell At a Time (OCAT): a unified framework to integrate and analyze single-cell RNA-seq data

**DOI:** 10.1186/s13059-022-02659-1

**Published:** 2022-04-20

**Authors:** Chloe X. Wang, Lin Zhang, Bo Wang

**Affiliations:** 1 University Health Network, Toronto, Canada; 2 Department of Statistical Sciences, University of Toronto, Toronto, Canada; 3 Department of Laboratory Medicine and Pathobiology, Temerty Faculty of Medicine, University of Toronto, Toronto, Canada; 4 Department of Computer Science, University of Toronto, Toronto, Canada; 5 Vector Institute, Toronto, Canada

**Keywords:** Integration, Single-cell RNA-seq, Differential gene expression, Trajectory inference, Pseudotime inference

## Abstract

Integrative analysis of large-scale single-cell RNA sequencing (scRNA-seq) datasets can aggregate complementary biological information from different datasets. However, most existing methods fail to efficiently integrate multiple large-scale scRNA-seq datasets. We propose OCAT, One Cell At a Time, a machine learning method that sparsely encodes single-cell gene expression to integrate data from multiple sources without highly variable gene selection or explicit batch effect correction. We demonstrate that OCAT efficiently integrates multiple scRNA-seq datasets and achieves the state-of-the-art performance in cell type clustering, especially in challenging scenarios of non-overlapping cell types. In addition, OCAT can efficaciously facilitate a variety of downstream analyses.

**Supplementary Information:**

The online version contains supplementary material available at (10.1186/s13059-022-02659-1).

## Background

The rapid advancement of transcriptome sequencing technologies in single cells (scRNA-seq) has witnessed the exponential growth in the number of large-scale scRNA-seq datasets. Integration of multiple scRNA-seq datasets from different studies has the great potential to facilitate the identification of both common and rare cell types. Batch effect correction is one of the biggest challenges when integrating multiple scRNA-seq datasets. Batch effect is the perturbation in measured gene expressions, often introduced by factors such as library preparation, sequencing technologies, and sample origins (donors). Batch effect is therefore likely to confound with true biological signals, resulting in the misclassification of cells by experiment rather than by their true biological identities. Batch effect removal has thus become a common practice prior to data integration, which introduces additional computational challenges. Most existing batch effect removal procedures assume that the biological effect is orthogonal to the batch effect, which is unlikely to be true in real life. Moreover, as the scale of the datasets increases, integrating multiple large-scale scRNA-seq datasets can introduce heavy, or sometimes unbearable, computational and memory storage burden.

Most of the existing scRNA-seq integration methods require explicit batch removal steps. One of the most commonly used approaches is mutual nearest neighbors (MNNs) [1], which employs paired cells (or MNNs) to project the data onto a shared subspace. However, this approach requires large runtime memory and long computation time to search for MNNs in the high dimensional space of gene expressions. Though some derivatives of the MNN method [2, 3] have attempted to improve memory efficiency by performing dimension reduction in the gene expression space, memory usage is still demanding when the number of single cells is large. Another common approach, Seurat [4], projects scRNA-seq data to a canonical correlation analysis (CCA) subspace and computes MNNs in the CCA subspace to correct batch effect. On the other hand, Harmony [5] iteratively removes batch effects after projecting scRNA-seq data to a principal component analysis (PCA) subspace. Harmony can also consume large memory when the number of cells is large. To meet these challenges in scRNA-seq integration, we hereby propose OCAT (One Cell At a Time), a fast and memory-efficient machine learning-based method that does not require explicit batch effect removal in integrating multiple scRNA-seq datasets. OCAT utilizes sparse encoding to integrate multiple heterogeneous scRNA-seq datasets, achieving state-of-the-art or comparable performance compared to existing methods.

OCAT offers three major advantages over existing methods. First, OCAT identifies hypothetical “ghost” cells of each dataset and constructs a sparse bipartite graph between each cell and the “ghost” cells, generating a sparsified encoding of each cell optimized for computational efficiency (*O*(*N*)). Secondly, by connecting each individual cell to the “ghost” cell set from all datasets, OCAT manages to capture the global similarity structure between single cells and thus does not require any explicit batch effect correction. Thirdly, the OCAT sparse graph encoding can be effectively transformed into cell feature representations that readily tackle a wide range of downstream analysis tasks, providing a unified solution to common single-cell problems such as differential gene expression analysis, trajectory inference, pseudotime inference, and cell type inference.

## Results

### The OCAT framework overview

OCAT integrates multiple large-scale scRNA-seq datasets using sparse encoding as the latent representations of the single-cell gene expressions. Given multiple scRNA-seq gene expression matrices as input, OCAT first identifies hypothetical “ghost” cells, centers of small cell neighborhoods, from each dataset. OCAT here only uses the *K*-means clustering algorithm to identify the feature representations of the “ghost” cells, but no cluster labels are assigned to the cells. OCAT next constructs a bipartite graph between all single cells and the “ghost” cell set using similarities as edge weights. OCAT further amplifies the strong connections and trims down the weak edges, by only retaining the edges connecting to the *s* most similar “ghost” cells for each single cell. We employ the local anchor embedding (LAE) algorithm [6] to further optimize the edge weights from each single cell to the remaining *s* most similar “ghost” cells, such that the resulting sparsified weights can most effectively reconstruct the transcriptomic features of the single cell. OCAT lastly captures the global cell-to-cell similarities through message passing between the “ghost” cells, which maps the sparsified weights of all single cells to the same global latent space. These transformed weights are then treated as the sparse encoding of each single cell.

**Fig. 1:**
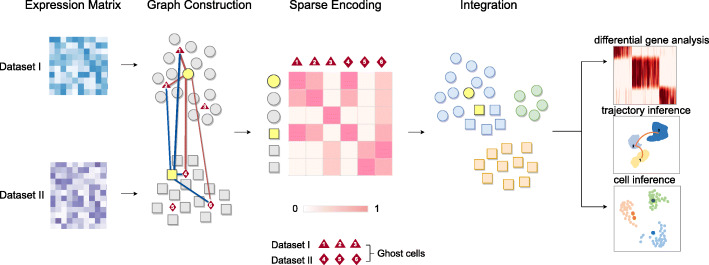
Schematic workflow of OCAT. When integrating multiple scRNA-seq datasets, OCAT first identifies “ghost” cells, centers of small cell neighborhoods, in each dataset. OCAT next constructs a bipartite graph connecting each cell to its most similar “ghost” cells. The edge weights connecting each cell’s closest “ghost” cells are treated as its OCAT sparse encoding. The OCAT sparse encoding can effectively correct the batch effect and facilitate various downstream analysis tasks, such as cell clustering, differential gene expression analysis, trajectory inference, and cell type inference

As the number of the most similar “ghost” cells *s* is much smaller than the number of genes, the OCAT latent representation is very sparse. We show that this sparse encoding can effectively facilitate downstream analyses, such as cell type clustering, differential gene expression analysis, trajectory and pseudotime inference, as well as cell type inference. Figure 1 outlines the workflow of the OCAT integration procedures and its various downstream analysis functionalities.

### Sparse encoding of single-cell transcriptomics effectively corrects batch effect and integrates multiple scRNA-seq datasets

When integrating multiple heterogeneous scRNA-seq datasets, most existing integration methods require iterations of explicit batch effect correction steps between every pair of datasets. Another common assumption in scRNA-seq data integration is that cell types are shared across all datasets, which is rarely true in real life. Such requirements and assumptions pose major challenges in the performance as well as computational efficiency to most existing integration methods. OCAT captures the global cell-to-cell similarity across datasets by connecting each single cell to the “ghost” cell set. OCAT thus does not require any explicit batch effect correction step and proves to be robust in identifying non-overlapping cell types unique to some datasets. The sparsified encoding also greatly accelerates the computational speed and reduces considerable amount of the memory usage when integrating multiple large-scale datasets.

**Fig. 2:**
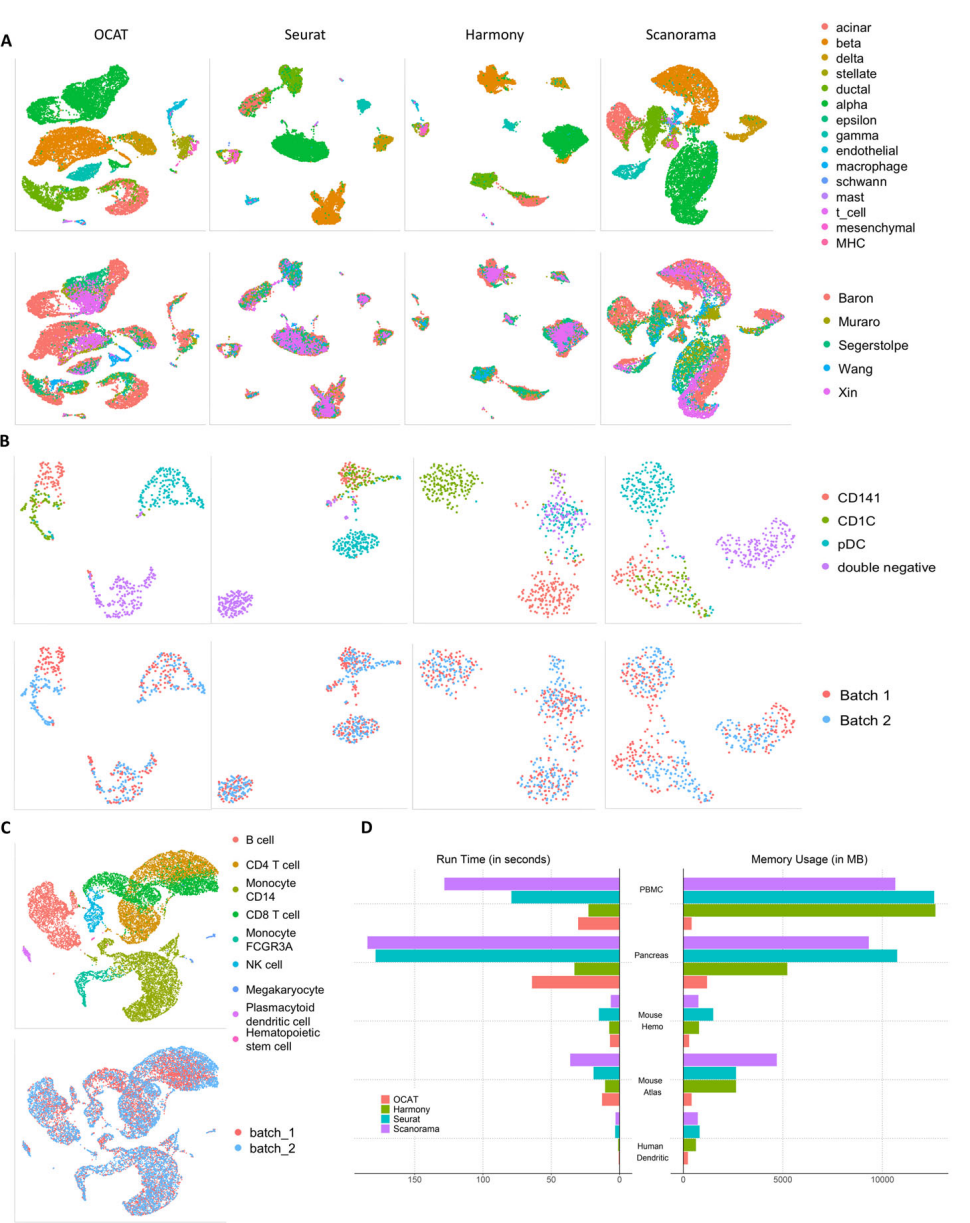
Integrating multiple scRNA-seq datasets with OCAT. **A** UMAP projection of five integrated human pancreatic scRNA-seq datasets from heterogeneous sequencing platforms by OCAT, Seurat v3, Harmony, and Scanorama. The top panel is colored by the annotated cell types, and the bottom panel is colored by the dataset. **B** UMAP projection of two integrated human dendritic datasets with non-overlapping cell types by OCAT, Seurat v3, Harmony, and Scanorama. The top panel is colored by the annotated cell types, and the bottom panel is colored by the dataset. **C** UMAP projection of two integrated PBMC scRNA-seq datasets by OCAT (see Additional file 3: Fig. S1 for a full comparison with Seurat v3, Harmony, and Scanorama). **D** Memory usage and runtime of OCAT, Seurat v3, Harmony, and Scanorama on five scRNA-seq integration tasks

One common assumption of existing integration methods is that any cell type present in one dataset must also be present in all the other datasets. We hereby demonstrate that if some non-overlapping cell types exist, methods that require such assumption can misclassify these cell types and confound biological effects with batch effects. The human dendritic dataset [7] consists of human blood dendritic cell (DC), namely, CD1C DC, CD141 DC, plasmacytoid DC (pDC), and double-negative cells. Tran et al. [8] further processed and manually split the data into two batches: batch 1 contains 96 pDC, 96 double-negative, and 96 CD141 cells, while batch 2 has 96 pDC, 96 double-negative, and 96 CD1C cells. CD141 cells are only present in batch 1, while CD1C cells are only present in batch 2. The visualization of cell type clustering in Fig. 2B shows that Seurat v3 [4], Harmony [5], and Scanorama [2] all falsely group CD141 and CD1C together. On the other hand, OCAT manages to distinguish CD141 and CD1C as two separate cell clusters. This verifies that the OCAT sparse encoding successfully recovers global cell-to-cell similarity across batches and captures true cell type identities by constructing the single cell to “ghost” cell bipartite graph. The cell type clustering metrics also reflect the same result, where OCAT has NMI_cell type_=0.7718, higher than all the other benchmarked methods (see Table 1 for a detailed comparison).

**Table 1:** Cell type clustering and batch correction performance on integrating multiple scRNA-seq datasets. The clustering performance is measured by the normalized mutual information (NMI), where NMI_cluster_=1 implies correct clustering by cell type annotations. (1−NMI_batch_)=1 implies no batch effect present after integration. Hyperparameter *m* is the number of “ghost” cells, and *d* is the dimension of the reduced gene expression matrix. The bolded entries are the best performance metrics for each scenario. See the “Methods” section for details on NMI_cluster_ and NMI_batch_ and Additional file 2: Table S1 for additional evaluation metrics

Dataset	*n*_cells_	*n*_genes_	*m*	*d*	Metric	OCAT	Seurat	Harmony	Scanorama
Human dendritic	576	16,594	20	80	NMI_cluster_	**0.7718**	0.7375	0.7653	0.7212
					1- NMI_batch_	0.9999	0.9999	**1.0000**	0.9999
Mouse atlas	6,954	5,558	45	70	NMI_cluster_	**0.8006**	0.6981	0.7625	0.6960
					1- NMI_batch_	**0.9999**	0.9970	0.9959	0.9905
Human pancreas	14,767	15,558	65	60	NMI_cluster_	**0.7949**	0.7947	0.7302	0.7249
					1- NMI_batch_	0.9650	**0.9762**	0.9600	0.9538
PBMC	15,476	33,694	40	120	NMI_cluster_	0.7424	**0.7932**	0.7851	0.7141
					1- NMI_batch_	0.9934	0.9943	**0.9944**	0.9939
Mouse hematopoietic	4,649	3,467	30	70	NMI_cluster_	0.5019	0.4606	0.4111	**0.5160**
					1- NMI_batch_	0.9648	0.9673	**0.9734**	0.9674

We then demonstrate the performance and efficiency of OCAT on integrating more than two large-scale heterogeneous scRNA-seq datasets. The pancreatic dataset consists of five human pancreatic scRNA-seq datasets sequenced with four different technologies (inDrop [9], CEL-Seq2 [10], Smart-Seq2 [11], SMARTer [12, 13]). Datasets generated by different sequencing platforms and technologies have inherent technical differences [14, 15], posing greater challenge to the integration task as the distributions of gene expressions vary significantly across the five datasets. Another challenge lies in the computational cost and memory consumption of integrating five datasets, caused by the iterative batch correction process for large number of cells with high dimensional gene expressions. Nevertheless, OCAT outperforms the other methods in correctly identifying the cell types without any highly variable gene selection or batch effect correction steps. Following the data pre-processing procedures outlined in Tran et al. [8], we integrate the five pancreatic datasets using OCAT, and benchmark with Seurat v3, Harmony, and Scanorama. The UMAP projection in Fig. 2A demonstrates that OCAT outperforms the other methods in correctly clustering the single cells (NMI_cell type_=0.7949), while achieving comparable batch correction performance (1−NMI_batch_=0.9650) (see Table 1 for details). We show in Fig. 2D that OCAT is more computationally and memory efficient than the other benchmarked methods. Notably, OCAT takes less than half of the runtime of Seurat v3 and Scanorama. Though Harmony runs slightly faster than OCAT, it consumes four times more memory than OCAT. Seurat v3 and Scanorama both require more than 8 times memory of OCAT.

We also validate the performance of OCAT on integrating mouse cell atlas [16], human peripheral blood mononuclear cell (PBMC) [13] and mouse hematopoietic stem and progenitor cell [17] datasets. OCAT achieves state-of-the-art or comparable performance with the other benchmarked methods (see Table 1, Fig. 2C, and Additional file 3: Fig. S1-3 for details). Notably, when integrating the two PBMC datasets with a total of 15,476 cells and 33,694 genes, OCAT is twice faster than Seurat v3 and three times faster than Scanorama. In addition, Harmony and Seurat v3 consume more than 29 times of OCAT’s memory usage, while Scanorama consumes more than 24 times of OCAT’s memory usage (Fig. 2D).

### OCAT unifies various downstream biological inferences

We demonstrate in this section that the OCAT sparse encoding can effectively facilitate various downstream analyses with important biological implications, such as cell type inference, differential gene expression analysis, and trajectory inference and pseudotime inference.

#### Sparse encoding of individual scRNA-seq dataset

**Fig. 3:**
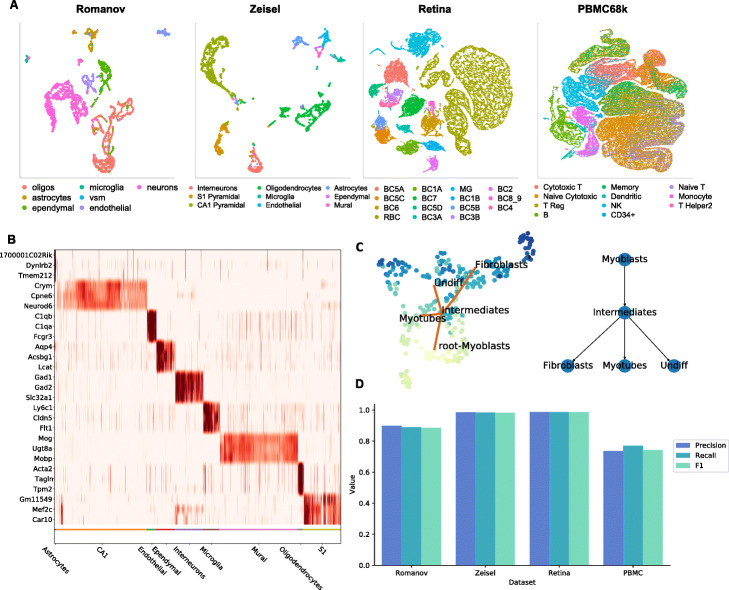
OCAT on individual scRNA-seq datasets. **A** UMAP projection of the OCAT sparsified embeddings for Romanov, Zeisel, Retina, PBMC 68k datasets, colored by the annotated cell types. **B** Differential gene expression analysis with OCAT on the Zeisel dataset. **C** OCAT trajectory inference and pseudotime inference on the HSMM dataset. **D** OCAT cell type inference performance on individual datasets Romanov, Zeisel, Retina, and PBMC, evaluated by three classification metrics, precision, recall, and F1

We first demonstrate that the OCAT sparse encoding framework can also be used to extract the latent representations of single cells in individual scRNA-seq datasets. For an individual scRNA-seq dataset, OCAT first identifies the “ghost” cells in the dataset, and constructs a bipartite graph that connects each single cell to the “ghost” cells. We demonstrate that OCAT efficiently embeds individual scRNA-seq datasets, with four large-scale datasets: Romanov [18], Zeisel [19], Retina [20], and PBMC 68k [21]. We show in Table 2 that OCAT consistently outperforms scVI [22], Seurat v3 [4], and SIMLR [23] in cell type clustering (see Fig. 3A for the UMAP visualizations based on the OCAT sparse encoding).

**Table 2:** Clustering performance of OCAT on four individual scRNA-seq datasets: Romanov, Zeisel, Retina and PBMC 68k, benchmarked with scVI, Seurat v3 and SIMLR. The clustering performance is measured by the Normalized Mutual Information (NMI), where NMI=1 implies correctly clustering all the cells with the same cell types while NMI=0 indicates random guessing. The bolded entries are the best performance metrics for each scenario. See Additional file 2: Table S5 for additional evaluation metrics

Dataset	*n*_cells_	*n*_genes_	*m*	*d*	OCAT	scVI	Seurat	SIMLR
Romanov	2,881	24,341	20	30	**0.6443**	0.5436	0.6343	0.4234
Zeisel	3,005	19,972	50	30	**0.7884**	0.7126	0.6724	0.7373
Retina	19,829	13,166	60	60	**0.8742**	0.7572	0.7865	0.6337
PBMC 68k	68,579	1000	40	40	**0.5750**	0.4638	0.4899	0.5344

#### Cell type inference

OCAT supports immediate and accurate cell type inference of incoming data, without repeating feature extraction procedures by combining the incoming data with the existing database. We denote the existing scRNA-seq datasets as the “reference” dataset, and the incoming unlabeled data as the “inference” dataset. For the reference dataset, OCAT first identifies a set of “ghost” cells and extracts the sparse features to train a support vector machine (SVM) [24] with the annotated cell types. For the inference cells, OCAT computes their sparse encoding using the pre-identified reference “ghost” cell set and transfers cell type labels to the incoming inference cells using the pre-trained SVM.

We first demonstrate OCAT’s cell type inference performance on four individual scRNA-seq datasets, Romanov [18], Zeisel [19], Retina [20], and PBMC 68k [21]. Each dataset is randomly split into 90% reference set and 10% inference set. The OCAT-extracted features of the inference cells based on the reference “ghost” cells yield high accuracy in cell type assignment (see Fig. 3D and Additional file 2: Table S5 for details). We then demonstrate that OCAT can infer cell types in a more challenging scenario across two heterogeneous scRNA-seq datasets. With two PMBC scRNA-seq datasets [3], we split each dataset into 90% reference set and 10% inference set. OCAT assigns cell types to the 10% inference set from dataset 2 based on the 90% reference set from dataset 1, and vice versa, achieving an F1 score of 0.8907 and 0.7719, respectively. We also conduct cell type inference experiments on two mouse atlas datasets [16, 25] and two human dendritic datasets [7, 8], both achieving high accuracy in cell type assignment (see Additional file 3: Fig. S5 for details).

We further explore the efficacy of OCAT when using multiple datasets as integrated reference. We integrate three Pancreas datasets, Baron [9], Muraro [10], and Segerstolpe [11], to obtain the combined OCAT features from 12,818 pancreatic cells as the reference set. We validate OCAT’s cell type inference performance on the first inference set Xin [13] with 1,492 cells, which yields a highly competitive F1 score of 0.9847. We then examine a more challenging inference set [12], as the inference cell distribution is known to deviate from the reference set. We highlight that OCAT effectively mitigates the distribution differences and achieves an F1 score of 0.8932 (see Additional file 2: Table S9 for details).

OCAT’s cell type inference functionality serves as an adaptive toolkit for biologists to leverage their existing reference datasets and annotate cell types of new incoming data. OCAT consistently demonstrates competitive cell type annotation performance despite the batch or technology differences between the reference and inference sets, which shows that it effectively mitigates batch effects. Moreover, OCAT’s integrative reference building functionality powered by the online learning algorithm allows the biologists to iteratively build their in-house cell atlases, as well as to connect with other available large-scale cell atlases in a fast and memory efficient way.

#### Differential gene expression analysis

Differential gene expression analysis is one of the most common approaches to facilitate cell type annotations. OCAT effectively selects the marker genes for each cell group based on the raw gene expression data. We demonstrate the efficacy of OCAT in differential gene expression analysis using the Zeisel dataset [19] that classifies 9 cell types in the mouse somatosensory cortex and hippocampal CA1 region. Figure 3B plots the top 3 marker genes for each cell type. OCAT manages to replicate the marker gene findings reported by Zeisel et al. [19], for example, *Gad1* and *Gad2* genes for interneuron cells and *Acta2* gene for mural cells. We further compare the top selected differential genes by OCAT with those identified by Seurat v3 [4] and show that the top selected genes are highly consistent between the two methods. For example, for the CA1 cell population, OCAT identifies *Crym, Cpne6, Neurod6, Gria1*, and *Wipf3* as the top five differential genes, and four of them are also in the top five differential genes selected by Seurat v3. We further show that the top feature genes identified by OCAT and Seurat v3 are also highly consistent for the other cell populations (see Additional file 2: Table S6 and Additional file 3: Fig. S6 for details).

In the demonstration with the Zeisel dataset, we use the annotated cell type labels to show OCAT’s accuracy to identify the most differentially expressed genes and thus facilitate cell type annotation. We show in Additional file 3: Fig. S9-11 that the OCAT predicted labels can also effectively identify the top 20 most differentially expressed genes and are robust to hyperparameter selection (see the “Hyperparameter sensitivity analysis in downstream tasks” section for details). Unlike the traditional differential gene expression analysis methods, OCAT does not impose any distributional assumptions on the expressions of genes and does not require gene selection or screening [26]. We demonstrate that OCAT’s differential gene analysis achieves comparative performance as existing methods, while being fast and memory-efficient with fewer assumptions.

#### Trajectory and pseudotime inference

OCAT is able to reconstruct the developmental trajectory and pseudotime of cells based on their transcriptomic profiles. In most cell populations, there exists a gradient of differentiation underlying the process of cell renewal, from progenitor cells to the terminally differentiated cell types. Based on the similarities in gene expressions, trajectory and pseudotime analyses infer the differentiation status of the cell types as well as individual cells. Trajectory inference first maps out the developmental lineages from the least differentiated to most differentiated cell types. Pseudotime analysis then orders the individual cells along the predicted lineages and assigns each cell a pseudotime, indicating its time stamp in the process of differentiation.

OCAT extracts a reduced “ghost” neighborhood graph between cell types by aggregating cell-to-cell similarities in each cluster. OCAT then infers the lineages by constructing the minimal spanning tree [27] over the aggregated “ghost” neighborhood graph that connects all the cell types. The least differentiated cell type is considered as the root cluster, which determines the unique directionality of the inferred lineages (see the “Methods” section for details). Lastly, to compute the pseudotime of each cell, OCAT appoints the least differentiated cell in each “ghost” neighborhood as the root cell. Traversing down the lineages, OCAT uses the root cell as the point of reference in each local neighborhood to assign pseudotime to individual cells.

We validate the performance of OCAT trajectory and pseudotime inference using the human skeletal muscle myoblast (HSMM) dataset [28]. The HSMM dataset contains time-series scRNA-seq data outlining the early stages of myogenesis. The 271 myoblast cells were collected at 0, 24, 48, and 72 hours of differentiation, with gold standard annotations based on known gene markers [28, 29]. OCAT infers the differentiation trajectory from myoblast to intermediate cells, followed by three separate branches into myotubes, fibroblasts, and undifferentiated cells. Fibroblasts and undifferentiated cells represent the two cell groups that exit the differentiation cycle prior to myotube formation. The inferred trajectory is consistent with the known biology of myotube formation as well as the original findings in Tran and Bader [29]. The pseudotime assigned by OCAT is highly correlated with the collection time stamps, with a Pearson correlation of 0.8743 by annotated cell type group. Additionally, following the procedures in Saelens et al. [30], we compare OCAT with Slingshot [31], PAGA Tree [32], and Monocle ICA [33] on trajectory and pseudotime inference with 28 gold standard real datasets using the dynverse R package [30]. OCAT is competitive in accurately assigning cell positions along the lineages as well as assisting downstream tasks of identifying important genes specific to the trajectory (see the “Methods” section, Additional file 3: Fig. S7 and Additional file 2: Table S7 for details).

OCAT’s trajectory and pseudotime inference functionalities allow biologists to infer developmental lineages and pseudotime based on transcriptomic similarity. These inferred developmental lineages outline the potential relationships between the cell clusters and thus facilitate the discovery of intermediate cell types and states that are critical in cell differentiation [28, 29].

## Discussion

In this work, we present OCAT, a unified machine learning framework for analyzing large-scale scRNA-seq datasets, which synergizes a wide range of downstream tasks crucial to biological discoveries. OCAT utilizes sparse encoding as the latent representation of single cell transcriptomics to amplify the true biological signals. Through the hypothetical “ghost” cells, the OCAT sparse encoding can capture the global cell-to-cell similarity across multiple heterogeneous datasets. We demonstrated that, without any batch effect correction, the sparse encoding of OCAT effectively separates the true biological differences among the cells from batch effects, achieving state-of-the-art performance with existing methods in cell type identification.

Unlike most existing methods, OCAT does not rely on highly variable gene selection to discriminate biological cell groups, which preserves the identities of non-overlapping cell types unique to some datasets. Furthermore, OCAT successfully leverages the high demand for computational resources in integrating large-scale scRNA-seq datasets. Most existing integration methods cluster single cells by computing global cell-to-cell adjacency matrix, which requires storing a *N*×*N* matrix and thus consumes large memory. On the other hand, OCAT uses the sparsified edge weights between each cell to the “ghost” cell set as its embedding. Because the number of “ghost” cells is way fewer than the number of genes, OCAT can scale up to integrate multiple scRNA-seq datasets with large number of cells and large number of genes, in a computational and memory efficient way.

OCAT identifies the hypothetical “ghost” cells, centers of small cell neighborhoods, using the *K*-means clustering algorithm. The goal here is not to assign cell type labels but to identify the “ghost” cells and obtain their feature representations, which are subsequently used to construct the bipartite graphs. After obtaining the sparse embedding of each cell, OCAT performs clustering algorithm to predict cell clusters. By default, OCAT uses the *K*-means clustering algorithm here, but this can be replaced by any clustering algorithms, such as Louvain and Leiden clustering algorithms.

The OCAT framework requires that the genes are aligned and shared across all the datasets, which is a common assumption for most existing integration methods. If some genes are missing for all cells in one dataset, OCAT cannot utilize these missing genes in its integration. However, with the advance of scRNA-seq technology, most signature genes are unlikely to be missing, which indicates that using the common genes shared across all the datasets should be sufficient to characterize the gene expressions of cells. If the reads of some cells are missing at random in one dataset, OCAT can easily adapt existing imputation methods (for example, *k*-nearest neighbor imputation) to “fill in” the missing values. If some major genes are systematically missing in one dataset (batch) but not in others, imputation may not work because imputation may be perfectly confounded with batch effect, which is a common limitation to existing integration methods.

OCAT effectively facilitates a variety of downstream analyses with important biological implications. For example, OCAT is readily applicable to analyzing individual scRNA-seq dataset, outperforming existing methods in cell type clustering. Moreover, OCAT can undertake challenging tasks such as differential gene expression analysis, trajectory inference, pseudotime inference, and cell type inference. For the trajectory inference analysis, OCAT assumes that cellular state of the same cell type cluster is similar. OCAT also assumes that only one root cluster (the least differentiated cluster) exists and produces more accurate prediction if the root cluster is known. For the pseudotime inference, we assume that the pseudotime values are similar along the trajectories. However, it has been pointed out that pseudotime sometimes may not necessarily reflect the true biological differentiation process.

## Conclusions

With its sparse encoding of scRNA-seq data, OCAT provides a fast and memory-efficient framework for analyzing and integrating large-scale scRNA-seq data. OCAT utilizes ghost cells to inform global cell-to-cell similarity, which effectively integrates data from multiple heterogeneous sources without highly variable gene selection or explicit batch effect correction. The OCAT package also offers a variety of built-in downstream analysis functionalities, such as differential gene selection, trajectory and pseudotime inferences, and cell type inference. With additional biological priors, OCAT has the great potential to better facilitate downstream analyses and extend to tackle more complex tasks such as cell-to-cell communication network inference, which we will explore as future work. OCAT is freely available at https://github.com/bowang-lab/OCAT[34].

## Methods

### The OCAT framework

OCAT endorses sparse encoding of the latent representations of the single-cell transcriptomics. Given multiple single-cell gene expression matrices as input, OCAT first identifies “ghost” cells from each dataset and connects each cell with all the ghost cells through a sparsified bipartite graph where the weights of the edges are treated as the encoding. OCAT then uses this sparse encoding to find the similarities between cells and to facilitate downstream analyses such as cell clustering, trajectory inference, cell type inference, and differential gene analysis. In the next sections, we outline the OCAT algorithms when integrating *H* heterogeneous scRNA-seq datasets, each consisting of *N*_1_,*N*_2_,…,*N*_*H*_ cells and *M* common genes. Additional file 2: Table S8 summarizes the notations.

### Data pre-processing

For the *h*th user-input raw gene expression matrix *R*_*h*_, OCAT first log-transforms each entry *r*_*ij*_ to:

1 $$  r'_{ij} = \text{log}_{10} (r_{ij}+1), \text{for}\ i=1, \ldots, N_{h}\ \text{and}\ j=1, \ldots, M,  $$

and normalizes $r^{\prime }_{ij}$ by:

2 $$ x_{ij} = \frac{r'_{ij}}{\sqrt{\sum_{i=1}^{N_{h}} r'_{ij}}}.  $$

The normalized gene expression vector for the *i*th single cell is ***x***_*i*_=(*x*_*i*1_,*x*_*i*2_,…,*x*_*iM*_)^*T*^, and the normalized gene expression matrix for the *h*th dataset is $X_{h} = \{\boldsymbol {x}_{i}\}_{i=1}^{N_{h}}$. We denote the normalized gene expression matrix for all *H* datasets as *X*.

### Dimension reduction of gene expression matrix

To efficiently encode the scRNA-seq datasets, OCAT further reduces the dimension of the normalized gene expression matrix *X* to *X*^′^. OCAT adopts the online fast similarity matching (FSM) algorithm [35] that projects each ***x***_*i*_ from *M*-dimensional to *d*-dimensional such that:

3 $$ \underset{\boldsymbol{x}'_{i} \in \mathbb{R}^{d}}{\text{min}} \sum_{k=1}^{N} \sum_{i=1}^{N} (\boldsymbol{x}_{k}^{T} \boldsymbol{x}_{k} - {\boldsymbol{x}'_{i}}^{T} \boldsymbol{x}'_{i})^{2},  $$

where ***x****i*′ is the low-dimensional representation of cell *i* and $N = \sum _{h=1}^{H} N_{h}$ is the total number of cells across all datasets. We denote $X = \{\boldsymbol {x}'_{i}\}_{i=1}^{N}$ as the low-dimensional representation for cells from all *H* datasets and $X_{h}' = \{\boldsymbol {x}'_{i}\}_{i=1}^{N_{h}}$ as the low-dimensional representation of the *h*th scRNA-seq dataset.

Though a vast collection of methods is available for dimension reduction, online FSM is much more efficient with a complexity of *O*(*NMd*) than the traditional principal component analysis (PCA) whose complexity is *O*(*M*^2^*N*+*M*^3^). When the number of genes, *M*, is large (ususally >20,000), online FSM is substantially faster than PCA. We recommend online FSM as the default option in the OCAT package but also offer PCA as an alternative option for the users.

### Identifying the “ghost” cells

OCAT introduces ghost cells to characterize the global similarity between single cells. Ghost cells are imaginary cells that are *K*-means cluster centers of *X*^′^, and they are not necessarily single cells from the input scRNA-seq datasets. For the *h*th dataset, OCAT identifies *m*_*h*_ ghost cells (*m*_*h*_*K*-means centers of $X^{\prime }_{h}$) each with *d*-dimensional features ***u***_*k*_, for *k*=1,2,…,*m*_*h*_. Let $U_{h} = \{\boldsymbol {u}_{k}\}_{k=1}^{m_{h}}$ be the representations of the ghost cells from the *h*th dataset. Then, the feature representations for all the ghost cells from *H* datasets are $U = \left (U_{1}^{T}, U_{2}^{T}, \ldots, U_{H}^{T} \right)^{T}$.

### Constructing sparsified bipartite graph

OCAT then constructs the bipartite graph that connects single cell one at a time, to all the ghost cells. The optimized edge weights to the nearest ghost cells are collectively treated as the sparsified representations of the cells. For the *i*th single cell (from any dataset), OCAT identifies its *s*_*h*_ closest ghost cells in the *h*th ghost cell set using cosine similarity, and denotes their indices as 〈*ih*〉. OCAT then optimizes the edge weights ***z***_〈*ih*〉_ using local anchor embedding (LAE) [6] by:

4 $$ \underset{\boldsymbol{z}_{\langle ih \rangle} \in \mathbb{R}^{s_{h}} }{\text{min}} \: \: \: \: \frac{1}{2} || \boldsymbol{x}'_{i} - U_{\langle ih \rangle} \boldsymbol{z}_{\langle ih \rangle} ||^{2}, \: \: \text{such that} \: \boldsymbol{1}^{T} \boldsymbol{z}_{\langle ih \rangle} = 1\ \text{and}\ \boldsymbol{z}_{\langle ih \rangle} \geq 0,  $$

where ***x****i*′ is the *d*-dimensional representation of cell *i*, and *U*_〈*ih*〉_ is the *d*-dimensional representation of its *s*_*h*_ closest ghost cells.

Similarly, we can obtain the optimized weights for cell *i* from all the ghost cell sets, denoted as *Z*_*i*,〈*i*〉_=(***z***_〈*i*1〉_,***z***_〈*i*2〉_,…,***z***_〈*iH*〉_)^*T*^. If a ghost cell is not one of the sh closest ghost cells to cell i, then the edge weight is reduced to 0, denoted as $Z_{i, \overline {\langle i \rangle }} = \boldsymbol {0}$. The collection of all the sparsified edge weights is $Z = \{\boldsymbol {z}_{i}\}_{i=1}^{N} \in \mathbb {R}^{N \times m}$, where $N = \sum _{h=1}^{H} N_{h}$ is the total number of single cells, and $m = \sum _{h=1}^{H} m_{h}$ is the total number of ghost cells.

### Message passing between single cells

OCAT further applies message passing algorithms [36] that connect the ghost cells among themselves. Based on the optimized edge weights *Z*, OCAT computes the ghost cell-to-ghost cell similarity *Z*^ghost^ by:

5 $$ Z^{\text{ghost}} = Z^{T} Z,  $$

and normalizes *Z*^ghost^ by:

6 $$ Z^{G} = D^{-\frac12} Z^{\text{ghost}} D^{-\frac12},  $$

where *D* is a diagonal matrix with:

7 $$ D_{kk} = \sum_{i=1}^{m} Z^{\text{ghost}}_{ik}, \text{for}\ k = 1, \ldots, m.  $$

With the normalized ghost cell-to-ghost cell similarity *Z*^*G*^, OCAT next obtains the refined sparse edge weights for each cell:

8 $$ Z^{W} = Z Z^{G}.  $$

For the edge weights of single cell *i*, $\boldsymbol {z}_{i}^{W}$, we normalize it by:

9 $$ \boldsymbol{z}_{i}^{\text{norm}} = \frac{\boldsymbol{z}_{i}^{W}}{\sqrt{\boldsymbol{z}_{i}^{W} \cdot \boldsymbol{z}_{i}^{W}}},  $$

and $\boldsymbol {z}_{i}^{\text {norm}}$ is the final sparse representation of cell *i*. We denote the final OCAT sparse embedding for all cells as:

10 $$ Z^{\text{norm}} = \{\boldsymbol{z}_{1}^{\text{norm}}, \boldsymbol{z}_{2}^{\text{norm}}, \ldots, \boldsymbol{z}_{N}^{\text{norm}} \},  $$

where $N= \sum _{h=1}^{H} N_{h}$ and $m = \sum _{h=1}^{H} m_{h}$.

For individual datasets, OCAT follows similar procedures as described above by setting *H*=1.

### Differential gene expression analysis

OCAT offers the functionality to find the differential genes for each cell type clusters. Denote the normalized gene expression matrix as *X*={*x*_*ij*_}, where *i*=1,…,*N* and *j*=1,…,*M*. For cell type cluster *C*, we compares the gene expression of cell type cluster *C* with all the other types, and we rank the top differentially expressed genes by the magnitude of:

11 $$ T_{j}^{C} = \frac{\left(\frac{1}{n_{C}} \sum \limits_{i \in C} x_{ij} - \frac{1}{N - n_{C}} \sum \limits_{i \not \in C} x_{ij} \right)^{2}}{\frac{1}{N} \sum \limits_{i=1}^{N} (x_{ij} - \bar{x}_{j})^{2} },  $$

where $\bar {x}_{j} = \sum _{i=1}^{N} x_{ij}/N $.

### Cell type inference

OCAT supports immediate cell type inference of incoming data based on existing databases, without re-computing the latent representations by combining the new incoming (“inference”) dataset and the existing (“reference”) dataset. Given an incoming inference set, OCAT first projects the normalized gene expression *X*_infer_ to the same *d*-dimensional subspace as the reference set, obtaining the reduced cell representation $X^{\prime }_{\text {infer}}$. OCAT then constructs a bipartite graph that connects these new inference cells to the ghost cells identified in the reference set following (4) and obtains the edge weights, *Z*_infer_, for the inference cells. The edge weights then go through the same message-passing procedures as the reference cells, resulting in $Z^{W}_{\text {infer}}$, the sparse representation of the new inference cells mapped to the same global subspace as the reference cells.

To assign cell type labels to the inference cells, OCAT trains a support vector machine (SVM) [24] based on the sparse representations of the reference cells, $Z^{W}_{\text {refer}}$, and the cell type labels for the reference cells. Based on the estimated coefficients from SVM, OCAT infers the cell type labels of the new incoming cells using $Z^{W}_{\text {infer}}$.

### Trajectory inference

Trajectory inference aims to computationally reconstruct the developmental trajectory of cells based on gene expressions. It outlines the temporal transition from the least differentiated to the most differentiated cell types. OCAT infers the developmental lineages by connecting the similarity graph between cell types with a minimum spanning tree [27].

Suppose we have an *N*×*m* dimensional gene expression embedding for the cells, for example, the sparse embedding by OCAT, *Z*^norm^. The cells are clustered into *c* cell types based on the embedding. OCAT computes the similarity score between cell type *p* and cell type *q*, *A*_*p*,*q*_, by averaging the pair-wise cell-to-cell cosine similarities between cell types *p* and *q*.

12 $$ A_{p,q} = \sum\limits_{u=1}^{n_{p}} \sum\limits_{v=1}^{n_{q}} a_{u, v}/n_{p} n_{q}, \hspace{0.15cm} \text{where}\ a_{u, v} = \frac{\boldsymbol{z}^{p}_{u} \boldsymbol{z}^{q}_{v} }{\Vert \boldsymbol{z}^{p}_{u} \Vert \Vert \boldsymbol{z}^{q}_{v} \Vert },  $$

$\boldsymbol {z}^{p}_{u}$ is the embedding vector for the *u*th cell in cell type *p*, $\boldsymbol {z}^{q}_{v}$ is the embedding vector for the *v*th cell in cell type *q*, and *n*_*p*_ and *n*_*q*_ are the number of cells in cell type *p* and *q*, respectively. ∥·∥ denotes the l2-norm.

Let $A = \{A_{p,q}\} \in \mathbb {R}^{c \times c}$ denote the matrix of pair-wise similarity scores between *c* cell types. OCAT constructs an undirected graph *G*^*C*^ from *A*, where each node represents a unique cell type, and the edge weight between two nodes (two cell types) is their similarity score. OCAT then obtains the minimum spanning tree *T* that connects all the nodes while minimizing the total sum of edge weights in the tree *T*. OCAT lastly adds directionality to the tree by taking the least differentiated cell type, namely, the root cell type, as the starting point of differentiation. Once the root cell type is determined, we obtain a unique directionality within the tree *T*.

### Pseudotime inference

Pseudotime analysis assigns each cell a time stamp along the lineages: less differentiated cells have earlier time stamps; more differentiated cells have later time stamps. Pseudotime provides more granularity to individual cells than the lineage ordering of cell types. OCAT defines a root cell in the root cluster, ***r***_1_, to serve as a reference to quantify differentiation. Biologically, ***r***_1_ represents the most primitive in the entire differentiation trajectory. OCAT identifies ***r***_1_ computationally by locating the cell whose spatial distances with other cells have the best accordance with the lineage ordering of cell types identified. OCAT then infers the extent to which a particular cell differentiates using its distance to the most primitive ***r***_1_, where less differentiated cells are closer to ***r***_1_, and vice versa.

To calculate the distance of the *u*th cell of type *p* to the first cell ***r***_1_, OCAT considers both the position of cluster *p* along the cell type lineages and the position of cell *u* in cluster *p*. We then define a root cell in every non-root cluster to serve as landmarks to connect the cell types along the lineages, denoted as $\boldsymbol {r}_{2}, \ldots, \boldsymbol {r}_{c} \in \mathbb {R}^{m}$. In a non-root cluster *p*, the cell with the closest average Euclidean distance with all cells in the previous cluster *p*−1 on the same lineage is assigned to be the root cell, ***r***_*p*_. OCAT defines a distance *D*_*i*_ for each cell in the dataset, where $D \in \mathbb {R}^{N}$. The distance for the *u*th cell in cluster *p* is defined as the sum of Euclidean distance between $\boldsymbol {z}^{p}_{u}$ and the current root cell cluster ***r***_*p*_, and the length of cell type lineages up to cluster *p*:

13 $$ D_{i} = \text{dis}(\boldsymbol{z}^{p}_{u}, \boldsymbol{r}_{1}) = \sqrt{\Vert \boldsymbol{z}^{p}_{u} - \boldsymbol{r}_{p} \Vert^{2}} + \sum_{l=2}^{p} \sqrt{\Vert \boldsymbol{r}_{l} - \boldsymbol{r}_{l-1} \Vert^{2}}.  $$

OCAT uses the normalized distance *D*_norm_ as the pseudotime measure:

14 $$ D_{\text{norm}} = \frac{D - \min(D)}{\max(D) - \min(D)}.  $$

### Evaluation metrics

#### Cosine similarity

We use cosine similarity as the similarity measure to identify the *s* closest “ghost” cells in constructing the sparse bipartite graph. The cosine similarity between single cell *i* and “ghost” cell *j* is defined as:

15 $$ \theta_{ij} = \frac{{\boldsymbol{x}'_{i}}^{T} \boldsymbol{u}_{j}}{\sqrt{{\boldsymbol{x}'_{i}}^{T} \boldsymbol{x}'_{i} \boldsymbol{u}_{j}^{T} \boldsymbol{u}_{j}}},  $$

where ***x****i*′ is the feature of the *i*th single cell, and ***u***_*j*_ is the feature of the *j*th “ghost” cell.

#### Clustering evaluation metrics

To assess the cell type clustering performance of the OCAT features, we compute the normalized mutual information (NMI) score of the predicted cell types and the annotated cell type labels (“ground truth”). The NMI score measures the overlap between the predicted cell type labels and true cell type labels normalized by entropy. We define entropy as:

16 $$  H(X) = -\sum \limits_{i=1}^{|X|}\frac{|X_{i}|}{N}\log \frac{|X_{i}|}{N},  $$

where |*X*| is the total number of categories, |*X*_*i*_| is the number of observations fall into category *i*, and *N* is the total number of observations.

The NMI score of cell type clustering is defined as:

17 $$  {}\text{NMI}_{\text{cluster}}(U,V) = \frac{2\text{MI}(U,V)}{H(U) + H(V)}, \: \: \text{where}\ \text{MI}(U,V) =\sum_{i=1}^{|U|} \sum_{j=1}^{|V|} \frac{|U_{i}\cap V_{j}|}{N} \log \frac{N|U_{i} \cap V_{j}|}{|U_{i}||V_{j}|},  $$

*U* is the set of predicted cell types, *V* is the set of annotated cell types (“ground truth”), *N* is the total number of cells, and *H*(·) is the entropy defined in (16). The value of NMI_cluster_ ranges from 0 to 1, where NMI_cluster_=1 indicates perfect clustering of single cells by the annotated cell types, and NMI_cluster_=0 indicates random guessing. In this work, we adopt the normalized_mutual_info_score function in the scikit-learn Python package [37] to compute the NMI scores.

#### Batch effect correction evaluation metric

OCAT does not impose explicit batch correction steps, but we demonstrate that OCAT is robust to batch effect by capturing the global similarities between single cells. To assess the batch effect correction performance of OCAT, we adopt a modified NMI metric as in (17), and define NMI_batch_ as:

18 $$ \text{NMI}_{\text{batch}}(I,J) = \frac{2\text{MI}(I,J)}{H(I) + H(J)},  $$

where *I* is the predicted batch labels, *J* is the true batch origin of each single cell, and MI is defined as in (17). NMI_batch_=0 indicates that the predicted labels are not confounded with the batch label, and NMI_batch_=1 indicates that the predicted labels are perfectly confounded with the batch labels. We report (1−NMI_batch_) such that a higher (1−NMI_batch_) value indicates better batch effect removal performance.

#### Other variations of clustering performance evaluation metrics

Besides the NMI score, we also assess the cell type clustering and batch effect correction performance using the adjusted mutual information (AMI) scores. The AMI score is defined as:

19 $$ \text{AMI}(U, V) = \frac{\text{MI}(U, V) - E(\text{MI}(U, V))}{\text{mean}(H(U), H(V)) - E(\text{MI}(U, V))},  $$

where *U* is the predicted label, *V* is the ground truth label, *E*(·) is the expectation, *H* is the entropy defined in (16), and MI is defined as in (17). We compute the AMI scores using the adjusted_mutual_info_score function in the scikit-learn Python package [37].

The Rand index (RI) is a measure of the percentage of correct decisions based on the true-positive (*tp*), false-positive (*fp*), true-negative (*tn*), and false-negative (*fn*) counts. The RI is defined as:

20 $$  \text{RI} = \frac{tp + tn}{tp + fp + fn + tn}.  $$

Here, we also adopt the adjusted Rand index (ARI) to assess the cell type clustering and batch effect correction. The ARI is defined as:

21 $$ \text{ARI} = \frac{\text{RI} - E(\text{RI})}{\max(\text{RI}) - E(\text{RI})},  $$

where RI is the Rand index defined in (20), and *E*(·) is the expectation. We compute the ARI scores using the adjusted_rand_score function in the scikit-learn Python package [37].

We report the AMI_cluster_,1−AMI_batch_,ARI_cluster_, and 1−ARI_batch_ metrics for all benchmarking integration datasets in Additional file 2: Table S1. We also report the AMI and ARI scores for clustering individual datasets in Additional file 2: Table S2.

#### Evaluation metric of trajectory and pseudotime inference

We benchmark OCAT with Slingshot [31], PAGA Tree [32], and Monocle ICA [33] on trajectory and pseudotime inference performance. We adopted the benchmarking procedure in dynverse [30]. Dynverse defines five categories (i.e., features, cell positions, neighborhood, topology, branch assignment) with 16 metrics to evaluate the performance of trajectory and pseudotime inference (see Additional file 2: Table S7 for the details of these metrics).

For the HSMM dataset, we also assessed the accuracy of OCAT pseudotime inference by reporting the Pearson correlation between the OCAT predicted pseudotime with the real time stamp labels by cell type cluster. The Pearson correlation *r* is defined as:

22 $$ r = \frac{\sum_{i=1}^{k}(u_{i} - \bar{u})(v_{i} - \bar{v})}{\sqrt{\sum_{i=1}^{k}(u_{i} - \bar{u})^{2} \sum_{i=1}^{k} (v_{i} - \bar{v})^{2}}},  $$

where *u*_*i*_ is the average inferred pseudotime of cells in cluster *i*, and *v*_*i*_ is the average real time stamp of cells in cluster *i*, with *i*∈{1,2,…,*k*} clusters. $\bar {u}$ is the average predicted pseudotime, and $\bar {v}$ is the average real time stamp. We used the pearsonr function in the scipy.stats Python package [37] to calculate the Pearson correlation score *r*.

#### Cell type inference evaluation metrics

To assess the performance of cell type assignment in cell type inference, we used the *precision*, *recall*, and *f*1 scores implemented in Python in the scikit-learn package [37]. We calculated the average of *precision*, *recall*, and *f*1 across all classes, weighted by the number of samples in each class (see details in Additional file 2: Table S5). The *precision*, *recall*, and *f*1 scores for each class are calculated from true positives (*tp*), false positives (*fp*), true negatives (*tn*), and false negatives (*fn*) in that particular class.

23 $$ \text{precision} = \frac{tp}{tp+fp}, \hspace{0.3cm} \text{recall} = \frac{tp}{tp + fn} \hspace{0.2cm} \text{and} \hspace{0.15cm} f1 = \frac{2 \times \text{precision}\times \text{recall}}{\mathrm{precision+recall}}.  $$

### Datasets

#### Integration datasets

We evaluated OCAT’s integration performance on five multi-source scRNA-seq datasets from Tran et al. [8]. Each dataset contains two or more batches of scRNA-seq data from different experiments or sequencing technologies for similar cell classes, presenting five different scenarios for batch correction.

- **Human dendritic dataset**: The human dendritic dataset contains two batches of scRNA-seq data sequenced using Smart-Seq2, with four cell populations identified, namely CD1C DC, CD141 DC, plasmacytoid DC, and double-negative cells [7]. Tran et al. [8] further removed cell type CD1C DC from batch 1 and cell type CD141 DC from batch 2, to create non-overlapping cell types among the batches. The resulting batch 1 contains 288 cells with three annotated cell types CD141 DC, double-negative, and plasmacytoid DC; batch 2 also contains 288 cells with three annotated cell types CD1C DC, double-negative, and plasmacytoid DC. The two batches share two common cell types plasmacytoid DC and double-negatives, with one unshared cell type respectively (CD141 DC and CD1C DC). This dataset presents a common scenario of batch effect with the presence of non-overlapping cell types.
- **Mouse atlas dataset**: The mouse atlas dataset contains two batches of scRNA-seq generated independently by Han et al. [16] using Microwell-Seq and the Tabula Muris Consortium [38] with 10x Genomics and Smart-Seq2 protocols. The 11 cell types with the highest cell numbers in both batches were retained. The resulting first batch contains the read counts of 4,239 cells, and the second batch contains 2,715 cells, with 15,006 common genes. This dataset captures the batch effect resulting from different sequencing technologies.
  The mouse atlas dataset contains the following 11 annotated cell types: T-cell, stromal, endothelial, macrophage, monocyte, epithelial, B-cell, neutrophil, dendritic, smooth-muscle, and NK. All cell types present in both batches.
- **Human pancreas dataset**: The human pancreas dataset consists of data from five different sources [9–13] of human pancreatic cells. Tran et al. [8] further processed the dataset by removing cells with ambiguous annotations, and the resulting batches contain a total of 14,767 cells with 15 different cell types. This dataset captures the batch effect across multiple sequencing technologies.
  In the resulting five batches, Baron et al. [9] contains 8,569 cells of 13 annotated cell types (acinar, beta, delta, stellate, ductal, alpha, epsilon, gamma, endothelial, macrophage, Schwann, mast, T cell); Muraro et al. [10] contains 2,122 cells of 9 annotated cell types (alpha, ductal, endothelial, delta, acinar, beta, gamma, mesenchymal, epsilon); Segerstolpe et al. [11] contains 2,127 cells of 11 annotated cell types (delta, alpha, gamma, ductal, acinar, beta, MHC, stellate, endothelial, epsilon, mast); Wang et al. [12] contains 457 cells of 7 annotated cell types (alpha, ductal, delta, beta, gamma, acinar, mesenchymal); and Xin et al. [13] contains 1,492 cells of 4 annotated cell types (beta, alpha, delta, gamma).
- **PBMC dataset**: The PBMC dataset contains two batches of PBMC data from healthy donors generated by 3’ and 5’ 10x Genomics protocols. A total of 8,098 cells from the 3’ batch and 7,378 cells from the 5’ batch were selected and annotated by Polanski et al. [3] with *k*-nearest neighbor clustering based on canonical markers. This dataset presents the challenge of integrating data with biological differences caused by sequencing protocols.
  The PBMC dataset contains 9 annotated cell types: CD4 T cell, CD8 T cell, monocyte CD14, B cell, NK cell, monocyte FCGR3A, plasmacytoid dendritic cell, megakaryocyte, and hematopoietic stem cell. All cell types exist in both batches.
- **Mouse hematopoietic dataset**: The mouse hematopoietic dataset contains two batches of mouse hematopoietic stem and progenitor cells, generated by Nestorowa et al. [17] with SMART-seq2 protocol and by Paul et al. [39] with MARS-seq protocol, respectively. Tran et al. [8] further extracted 2,729 well-annotated cells from the MARS-seq dataset. The resulting SMART-seq2 data contains 1,920 cells, and the MARS-seq data contains 2,729 cells, with 3,467 genes common genes retained.
  The MARS-seq batch contains 3 annotated cell types: GMP, MEP, and CMP. The SMART-seq2 batch contains 7 annotated cell types: MPP, MEP, CMP, LTHSC, LMPP, Unsorted, and GMP. This dataset therefore captures the batch effect from not only different sequencing technologies but also non-overlapping cell types.

#### Individual datasets

- **The Romanov dataset** [18] contains the RNA-seq data from 2,881 single cells with 24,341 genes in mouse hypothalamus obtained using Illumina HiSeq platform. Seven cell type clusters were identified by divisive biclustering method and annotated based on lineage-specific protogene markers.
  The Romanov dataset contains 7 annotated cell types: oligos, neurons, ependymal, astrocytes, endothelial, vsm, and microglia.
- **The Zeisel dataset** [19] contains the 3’-end counts of the unique molecular identifiers (UMI) assays from 3,005 single cells with 4,412 genes in the mouse somatosensory cortex and hippocampal CA1 region.
  The Zeisel dataset contains 9 annotated cell types: S1 and CA1 pyramidal neurons, interneurons, oligodendrocytes, astrocytes, microglia, vascular endothelial cells, mural cells, and ependymal cells.
- **The mouse retina dataset** [20] contains 19,829 single cells with 13,166 genes extracted from the Macoskco dataset (originally 44,808 cells), containing UMI (3’-end) counts obtained by Drop-seq.
  The mouse retina dataset contains 15 annotated cell types: BC1A, BC1B, BC2, BC3A, BC3B, BC4, BC5A, BC5B, BC5C, BC5D, BC6, BC7, BC8_9, MG, and RBC.
- **The PBMC 68k dataset** [21] contains the UMI (3’-end) counts from 68,579 peripheral blood mononuclear cells (PBMCs) with 1,000 genes profiled using the 10x Genomics GemCode platform.
  The PBMC 68k dataset contains 11 annotated cell types: CD14+ monocyte, CD19+ B, CD34+, CD4+ T Helper2, CD4+/CD25 T Reg, CD4+/CD45RA+/CD25-naive T, CD4+/CD45RO+ memory, CD56+ NK, CD8+ cytotoxic T, CD8+/CD45RA+ naive cytotoxic, and dendritic.
- **The human skeletal muscle myoblast (HSMM) dataset** [29] contains time-series RNA-seq data of 271 cells collected at 0, 24, 48, and 72 hours since human myoblast culture in the differentiation media. Five cell groups were annotated using GSVA based on known gene markers: myoblasts, intermediates, myotubes, fibroblasts, and undifferentiated.

### Hyperparameter selection and sensitivity analysis

The sparse encoding workflow of OCAT involves the specification of three hyperparameters: *d* as the projection dimension from the gene feature space by online FSM in the pre-processing step, *m* as the number of “ghost” cells each single cell connects to in the bipartite graph, and *s* as the closest “ghost” cells to construct the sparsified encoding of each cell. Here, we define *p*=*s*/*m* as the percentage of closet “ghost” cells chosen to embed the gene expression of each single cell. The number of closest “ghost” cells is computed as *s*=⌈*pm*⌉.

Here, we performed a set of sensitivity analyses to assess the impact of hyperparameter values on the OCAT sparse encoding in integrating multiple scRNA-seq data as well as clustering individual scRNA-seq data. We demonstrate that the OCAT sparse encoding is robust to various combinations of hyperparameters by comparing the cell type clustering performance.

#### Hyperparameter sensitivity analysis in integrating multiple datasets

We used the mouse atlas dataset as an example to demonstrate the robustness of OCAT embedding in integrating multiple datasets. We reported the NMI of cell type clustering using OCAT embeddings with *m*_1_=*m*_2_=45,*d*=70 and *p*=0.3 in Table 1, where *m*_1_ and *m*_2_ are the number of “ghost” cells selected for batch 1 and batch 2, respectively. We first set *m*_1_=*m*_2_=*m* and assessed the sensitivity of *d*, *m*, and *p* on the cell type clustering performance by taking the following hyperparameter values: *d*∈{40,50,60,70,80,90,100,110,120},*m*∈{20,25,30,35,40,45,50,55,60}, and *p*∈{0.1,0.3,0.5,0.7}. Additional file 3: Fig. S4A plots the NMI_cell type_ of each hyperparameter combination. The NMI values range from 0.7286 to 0.8007 with a median of 0.7809 and a standard deviation of 0.0144, which implies that the OCAT encoding is robust to the specification variations of *d*, *m*, and *p* in integrating multiple datasets.

We then assessed how the values of *m*_1_ and *m*_2_ impact the OCAT sparse encoding and subsequent clustering performance. We allow *m*_1_ and *m*_2_ to be different, taking values *m*_1_,*m*_2_∈{20,25,30,35,40,45,50,55,60}, while fixing *d*=70 and *p*=0.3. Additional file 3: Fig. S4C plots the NMI_cell type_ of each hyperparameter combination with a heatmap. The NMI values range from 0.7378 to 0.8007 with median 0.7812 and standard deviation 0.0162, which implies that the OCAT encoding is robust to the specification of the number of “ghost” cells in each batch.

#### Hyperparameter sensitivity analysis in single datasets

We next assessed the sensitivity of *d*, *m*, and *p* on clustering performance using the Zeisel dataset as an example. We reported the NMI of cell type clustering using OCAT embeddings with *m*=50,*d*=30, and *p*=0.3 in Table 2. Here, we assessed the sensitivity by taking the following hyperparameter values *d*∈{20,30,40,50,60,70,80,90,100},*m*∈{30,35,40,45,50,55,60,65,70}, and *p*∈{0.1,0.3,0.5,0.7}. Additional file 3: Fig. S4B plots the cell type clustering NMI metrics of each hyperparameter combination. Under each combination, the variation of the NMI metrics is minimal, where the NMI values range from 0.6572 to 0.7987 with a median of 0.7591 and a standard deviation of 0.0152. This demonstrates that the OCAT sparse encoding is robust to hyperparameter specifications in cell type clustering with single datasets.

#### Hyperparameter sensitivity analysis in downstream tasks

We further examined the impact of hyperparameter choices on downstream tasks, such as differential gene expression analysis. We used the Zeisel dataset to demonstrate the robustness of OCAT to the choices of *d*, *m*, and *p* in identifying the most differentially expressed genes. In the differential gene expression analysis carried out in this work, we reported the top differentially expressed genes in the Zeisel dataset using OCAT embedding with hyperparameters *d*=30,*m*=50, and *p*=0.3. Here, we assessed the sensitivity by taking the following hyperparameter values *d*∈{20,30,40,50,60,70,80,90,100},*m*∈{30,40,50,60,70}, and *p*∈{0.1,0.3,0.5,0.7}. We compared the top 20 genes selected in each parameter setting with the ones selected in the original analysis with *d*=30,*m*=50, and *p*=0.3. We showed that the top 20 selected genes are highly consistent across all parameter settings. We illustrated the number of overlapped genes in Additional file 3: Fig. S8, S9, and S10 for three sample cell types: CA1 pyramidal, S1 pyramidal, and interneurons.

#### Hyperparameter recommendations

OCAT has three hyperparameters, *d* (the dimension of the reduced gene expression matrix), *m* (the number of ghost cells), and *s* (the number of most similar ghost cells). We also reparameterize *s*=*p*∗*m*, where *p* represents the percentage of ghost cells selected to construct the sparsified embeddings. Users can fine-tune either *p* or *s* to adjust the sparsity of the OCAT embedding.

The choice of *d* should be based on the number of genes. In general, we recommend *d* to be around 1/100 times the number of genes, and the value of *d* should range from 30 to 150. The choice of *m* should be based on the number of cells in each dataset as well as the number of cell type clusters. We recommend *m* to be around 1/100 times the number of cells, and the value of *m* should range from 20 to 100. Another consideration when choosing *m* is the number of cell type clusters. We suggest increasing the value of *m* if the users desire more subclusters. For example, for a dataset with 5,000 cells, we suggest *m*=50. However, if the users would like to see more than 30 subclusters, *m* should increase to 60 or 70. The choice of *p* is based on the desired sparsity for the OCAT encoding. We have shown in the “Hyperparameter selection and sensitivity analysis” section that OCAT is robust to the choice of *p*. We recommend *p* is set to 0.3 (the default value) so that the OCAT embedding is sparse and computationally efficient.

We have shown in the “Hyperparameter selection and sensitivity analysis” section that the OCAT is robust to the choice of hyperparameter values in both clustering and downstream analysis. This general recommendation provides a guideline for users to navigate their exploratory analysis. For advanced users who wish to identify their desirable hyperparameter settings, OCAT offers a hyperparameter tuning functionality that examines multiple combinations of hyperparameter values and returns suitable evaluation metrics for advanced users (see Additional file 1: Notes 2.1 for details).

### Differential gene expression analysis and comparison

OCAT effectively identifies the differential genes for cell type annotation. We demonstrate the performance of OCAT differential gene expression analysis using the Zeisel dataset [19]. We summarize the top 5 OCAT selected differential genes in Additional file 2: Table S6 and show that OCAT manages to replicate the marker genes reported by Zeisel et al. [19], for example, *Gad1* and *Gad2* genes for interneuron cells and *Acta2* gene for mural cells.

We further compare the top differentially expressed genes for each cell type identified by OCAT and Seurat and report the number of top differentially expressed genes selected by both methods. Additional file 2: Table S6 summarizes the top 5 differential genes identified by both OCAT and Seurat, and the identified genes are highly consistent for most cell types. We also compare the top *k* differential genes identified by the two methods, and plot the number of overlapped genes (≤*k*) against *k*, the number of top differentially expressed genes, in Additional file 3: Fig. S6. The closer a dot to the diagonal line, the more overlap between the top differentially expressed genes selected by both methods. The plotted line for each cell type is close to the dotted diagonal line, which suggests that the top differentially expressed genes selected by OCAT and Seurat are highly consistent.

### Benchmarking specifics

#### Integrating multiple datasets

We benchmark the cell type clustering performance and batch correction performance of OCAT against Seurat v3 [4], Scanorama [2], and Harmony [5]. We followed the recommended settings and workflows of all the methods, with the specific parameters as follows. For Seurat v3, we followed the default settings, with no cells or genes screened out and selected 2000 most variable genes. We chose the top 50 principal components as the Seurat embeddings for the cells. To benchmark against Harmony, we adopted the Seurat object pre-processed as in the Seurat v3 benchmarking process, but extracted the top 50 features as the Harmony features using the RunHarmony function. For Scanorama, we added a manual step to normalize the raw scRNA-seq count matrix, and ran its recommended pipeline. We subsequently performed *k*-means clustering on the embeddings using the KMeans function and computed the NMI, AMI, and ARI for batch correction and cell type clustering metrics using the adjusted_rand_score, adjusted_mutual_info_score, normalized_mutual_info_score functions from the sklearn python package.

#### Individual scRNA-seq datasets

We benchmarked the cell type clustering performance of OCAT with Seurat v3 [4], scVI [22], and SIMLR [23]. For Seurat v3, we followed the default settings, with no cells screened out in the gene by cell count matrix. We chose the top 50 principal components as the Seurat features of the single cells. We adopt the Seurat default FindClusters function to cluster the cells. When benchmarking with scVI (version 0.6.5), we followed the default settings, and down-sampled 500 most variable genes out of all the genes available. We set the maximum number of epochs as 400, and used 90% of the dataset as the training set. We lastly used *k*-means to predict the clusters of the cells. We also benchmarked OCAT with SIMLR. SIMLR takes the same raw cell by gene matrix as OCAT. We adopted the SIMLR_Large_Scale function for all datasets. For the Zeisel dataset, we transformed the gene expression matrix *X* to log(*X*+1), and set the SIMLR tuning parameter *k*=50 and assessed the number of principal components *kk*=150. For the PBMC dataset, we used the raw cell by gene matrix and adopted the default SIMLR settings *k*=10 and *kk*=100. For the retina dataset, we used the raw cell by gene matrix and set the tuning parameter *k*=30 and *kk*=300. For the Romanov dataset, we used the raw cell by gene matrix and set the tuning parameter *k*=25 and *kk*=400. We performed *k*-means clustering on the embeddings using the KMeans function and computed the NMI, AMI, and ARI for cell type clustering metrics using the adjusted_rand_score, adjusted_mutual_info_score, normalized_mutual_info_score functions from the sklearn python package.

#### Benchmarking OCAT trajectory and pseudotime inference

We benchmarked OCAT’s trajectory and pseudotime inference performance with Slingshot [31], PAGA Tree [32], and Monocle ICA [33] using the dynverse R package on 28 gold standard real datasets [30]. The input datasets consist of expression matrices from a wide range of dynamic processes and sequencing technologies. The gold standard annotations on trajectory topologies were obtained from biological evidence besides the expression matrices, including four different trajectory types (i.e., linear, tree, multifurcation, bifurcation). We evaluated the trajectory and pseudotime assignment by each method using five aggregated metrics (features, cell positions, neighborhood, topology, branch assignment) derived from 16 dynverse metrics (see Additional file 2: Table S7 for details on metric aggregation and interpretation).

In OCAT’s benchmarking workflow, for each dataset, we first extracted the OCAT sparse encodings of the single cells, with hyperparameters *d* by package default and *m* selected based on the number of single cells. We subsequently inferred cluster labels by *k*-means clustering on the sparse encodings and then ran OCAT trajectory analysis to estimate the cell lineages. For pseudotime analysis, we used the root cluster inferred by OCAT in the previous trajectory step as input. For Slingshot [31], PAGA Tree [32], and Monocle ICA [33], we used the benchmarking pipeline provided by Saelens et al. [30] with dynverse-specified hyperparameters, which computes the lineages and pseudotime of the single cells in the reduced feature space for each dataset in a similar fashion.

### Runtime and memory usage

We benchmarked the runtime and memory usage of OCAT against Seurat v3 [4], Harmony [5], and Scanorama [2]. We timed the function call that performs data integration using the time.time() function in Python. We monitored the memory usage with HTOP and recorded the maximum memory usage occurred during the execution of each integration pipeline on a desktop workstation (Intel(R) Xeon(R) CPU @ 3.60 GHz processor).

## Supplementary Information

**Additional file 1** Supplementary notes on the online Fast Similarity Matching (FSM) and Local Anchor Embedding (LAE) algorithms used in OCAT.

**Additional file 2** Supplementary Tables S1-S9.

**Additional file 3** Supplementary Figures S1-S10.

**Additional file 4** Review history.

## Data Availability

All data can be freely downloaded and are described in the “Methods” section. The human dendritic dataset consists of human blood dendritic cells scRNA-seq data from GEO accession GSE80171 [7]. The dataset is further split into two batches by Tran et al. [8]. The mouse atlas datasets are from GEO under accession GSE108097 [16] and GSE109774 [38]. The human pancreas datasets are retrieved from GEO accession numbers GSE85241 [9], E-MTAB-5061 [10], GSE84133 [11], GSE83139 [12], and GSE81608 [13]. The PBMC dataset is available from Short Read Archive under accession number SRP073767 [21]. The mouse hematopoietic dataset was retrieved from GEO GSE81682 [17] and GSE72857 [39]. The Romanov dataset can be retrieved from GEO GSE74672 [18]. The Zeisel dataset can be retrieved from GEO GSE60361 [19]. The mouse retina dataset can be retrieved from GEO GSE81905 [20]. The PBMC 68k dataset can be retrieved from the Short Read Archive under accession number SRP073767 [21]. The human skeletal muscle myoblast dataset can be retrieved from GEO, accession number GSE52529 [28]. The OCAT python package and code used to analyze the data is deposited in Zenodo (doi: 10.5281/zenodo.6270539) [40] and is freely available with vignettes on the GitHub repository https://github.com/bowang-lab/OCAT under a GPLv3 license [34].

## References

[1] Haghverdi L, Lun AT, Morgan MD, Marioni JC (2018). Batch effects in single-cell rna-sequencing data are corrected by matching mutual nearest neighbors. Nat Biotechnol.

[2] Hie B, Bryson B, Berger B (2019). Efficient integration of heterogeneous single-cell transcriptomes using scanorama. Nat Biotechnol.

[3] Polański K, Young MD, Miao Z, Meyer KB, Teichmann SA, Park J-E (2020). Bbknn: fast batch alignment of single cell transcriptomes. Bioinformatics.

[4] Stuart T, Butler A, Hoffman P, Hafemeister C, Papalexi E, Mauck III WM, Hao Y, Stoeckius M, Smibert P, Satija R (2019). Comprehensive integration of single-cell data. Cell.

[5] Korsunsky I, Millard N, Fan J, Slowikowski K, Zhang F, Wei K, Baglaenko Y, Brenner M, Loh P-r, Raychaudhuri S (2019). Fast, sensitive and accurate integration of single-cell data with Harmony. Nat Methods.

[6] Liu W, He J, Chang S-F (2010). Large graph construction for scalable semi-supervised learning. ICML.

[7] Villani A-C, Satija R, Reynolds G, Sarkizova S, Shekhar K, Fletcher J, Griesbeck M, Butler A, Zheng S, Lazo S (2017). Single-cell rna-seq reveals new types of human blood dendritic cells, monocytes, and progenitors. Science.

[8] Tran HTN, Ang KS, Chevrier M, Zhang X, Lee NYS, Goh M, Chen J (2020). A benchmark of batch-effect correction methods for single-cell rna sequencing data. Genome Biol.

[9] Baron M, Veres A, Wolock SL, Faust AL, Gaujoux R, Vetere A, Ryu JH, Wagner BK, Shen-Orr SS, Klein AM (2016). A single-cell transcriptomic map of the human and mouse pancreas reveals inter-and intra-cell population structure. Cell Syst.

[10] Muraro MJ, Dharmadhikari G, Grün D, Groen N, Dielen T, Jansen E, van Gurp L, Engelse MA, Carlotti F, de Koning EJ (2016). A single-cell transcriptome atlas of the human pancreas. Cell Syst.

[11] Segerstolpe Å., Palasantza A, Eliasson P, Andersson E-M, Andréasson A-C, Sun X, Picelli S, Sabirsh A, Clausen M, Bjursell MK (2016). Single-cell transcriptome profiling of human pancreatic islets in health and type 2 diabetes. Cell Metab.

[12] Wang YJ, Schug J, Won K-J, Liu C, Naji A, Avrahami D, Golson ML, Kaestner KH (2016). Single-cell transcriptomics of the human endocrine pancreas. Diabetes.

[13] Xin Y, Kim J, Okamoto H, Ni M, Wei Y, Adler C, Murphy AJ, Yancopoulos GD, Lin C, Gromada J (2016). RNA sequencing of single human islet cells reveals type 2 diabetes genes. Cell Metab.

[14] Hicks SC, Townes FW, Teng M, Irizarry RA (2018). Missing data and technical variability in single-cell RNA-sequencing experiments. Biostatistics.

[15] Tung P-Y, Blischak JD, Hsiao CJ, Knowles DA, Burnett JE, Pritchard JK, Gilad Y (2017). Batch effects and the effective design of single-cell gene expression studies. Sci Rep.

[16] Han X, Wang R, Zhou Y, Fei L, Sun H, Lai S, Saadatpour A, Zhou Z, Chen H, Ye F (2018). Mapping the mouse cell atlas by microwell-seq. Cell.

[17] Nestorowa S, Hamey FK, Pijuan Sala B, Diamanti E, Shepherd M, Laurenti E, Wilson NK, Kent DG, Göttgens B (2016). A single-cell resolution map of mouse hematopoietic stem and progenitor cell differentiation. Blood J Am Soc Hematol.

[18] Romanov RA, Zeisel A, Bakker J, Girach F, Hellysaz A, Tomer R, Alpar A, Mulder J, Clotman F, Keimpema E (2017). Molecular interrogation of hypothalamic organization reveals distinct dopamine neuronal subtypes. Nat Neurosci.

[19] Zeisel A, Muñoz-Manchado AB, Codeluppi S, Lönnerberg P, La Manno G, Juréus A, Marques S, Munguba H, He L, Betsholtz C (2015). Cell types in the mouse cortex and hippocampus revealed by single-cell rna-seq. Science.

[20] Shekhar K, Lapan SW, Whitney IE, Tran NM, Macosko EZ, Kowalczyk M, Adiconis X, Levin JZ, Nemesh J, Goldman M (2016). Comprehensive classification of retinal bipolar neurons by single-cell transcriptomics. Cell.

[21] Zheng GX, Terry JM, Belgrader P, Ryvkin P, Bent ZW, Wilson R, Ziraldo SB, Wheeler TD, McDermott GP, Zhu J (2017). Massively parallel digital transcriptional profiling of single cells. Nat Commun.

[22] Lopez R, Regier J, Cole MB, Jordan MI, Yosef N (2018). Deep generative modeling for single-cell transcriptomics. Nat Methods.

[23] Wang B, Zhu J, Pierson E, Ramazzotti D, Batzoglou S (2017). Visualization and analysis of single-cell rna-seq data by kernel-based similarity learning. Nat Methods.

[24] Noble WS (2006). What is a support vector machine?. Nat Biotechnol.

[25] Consortium TM (2018). Single-cell transcriptomics of 20 mouse organs creates a tabula muris. Nature.

[26] Rapaport F, Khanin R, Liang Y, Pirun M, Krek A, Zumbo P, Mason CE, Socci ND, Betel D (2013). Comprehensive evaluation of differential gene expression analysis methods for RNA-seq data. Genome Biol.

[27] Kruskal JB (1956). On the shortest spanning subtree of a graph and the traveling salesman problem. Proc Am Math Soc.

[28] Trapnell C, Cacchiarelli D, Grimsby J, Pokharel P, Li S, Morse M, Lennon NJ, Livak KJ, Mikkelsen TS, Rinn JL (2014). The dynamics and regulators of cell fate decisions are revealed by pseudotemporal ordering of single cells. Nat Biotechnol.

[29] Tran TN, Bader GD (2020). Tempora: Cell trajectory inference using time-series single-cell RNA sequencing data. PLoS Comput Biol.

[30] Saelens W, Cannoodt R, Todorov H, Saeys Y (2019). A comparison of single-cell trajectory inference methods. Nat Biotechnol.

[31] Street K, Risso D, Fletcher RB, Das D, Ngai J, Yosef N, Purdom E, Dudoit S (2018). Slingshot: cell lineage and pseudotime inference for single-cell transcriptomics. BMC Genomics.

[32] Wolf FA, Hamey FK, Plass M, Solana J, Dahlin JS, Göttgens B, Rajewsky N, Simon L, Theis FJ (2019). Paga: graph abstraction reconciles clustering with trajectory inference through a topology preserving map of single cells. Genome Biol.

[33] Qiu X, Mao Q, Tang Y, Wang L, Chawla R, Pliner HA, Trapnell C (2017). Reversed graph embedding resolves complex single-cell trajectories. Nat Methods.

[34] Wang C, Zhang L, Wang B. OCAT: A unified framework to integrate and analyze single-cell RNA-seq data. GitHub. 2021. https://github.com/bowang-lab/OCAT. Accessed 07 Oct 2021. 10.1186/s13059-022-02659-1 PMC9019955 35443717

[35] Giovannucci A, Minden V, Pehlevan C, Chklovskii DB (2018). Efficient principal subspace projection of streaming data through fast similarity matching. 2018 IEEE International Conference on Big Data (Big Data).

[36] Pearl J (1988). Probabilistic reasoning in intelligent systems: networks of plausible inference.

[37] Buitinck L, Louppe G, Blondel M, Pedregosa F, Mueller A, Grisel O, Niculae V, Prettenhofer P, Gramfort A, Grobler J, Layton R, VanderPlas J, Joly A, Holt B, Varoquaux G (2013). API design for machine learning software: experiences from the scikit-learn project. ECML PKDD Workshop: languages for data mining and machine learning.

[38] Schaum N, Karkanias J, Neff NF, May AP, Quake SR, Wyss-Coray T, Darmanis S, Batson J, Botvinnik O, Chen MB (2018). Single-cell transcriptomics of 20 mouse organs creates a tabula muris: the Tabula Muris Consortium. Nature.

[39] Paul F, Arkin Y, Giladi A, Jaitin DA, Kenigsberg E, Keren-Shaul H, Winter D, Lara-Astiaso D, Gury M, Weiner A (2015). Transcriptional heterogeneity and lineage commitment in myeloid progenitors. Cell.

[40] Wang C, Zhang L, Wang B. OCAT: a unified framework to integrate and analyze single-cell RNA-seq data. Zenodo. 2022. 10.5281/zenodo.6270540. 10.1186/s13059-022-02659-1 PMC9019955 35443717

