# Supplementary material for: One Cell At a Time (OCAT): a unified framework to integrate and analyze single-cell RNA-seq data
Source: Genome Biol. 2022 Apr 20;23:102. doi: 10.1186/s13059-022-02659-1 (PMC9019955; doi:10.1186/s13059-022-02659-1)
Supplement: Supplementary file 1 — Additional file 1 Supplementary notes on the online Fast Similarity Matching (FSM) and Local Anchor Embedding (LAE) algorithms used in OCAT. [file 13059_2022_2659_MOESM1_ESM.pdf]

One Cell At a Time – Supplementary Material

Chloe Wang, Lin Zhang, Bo Wang

1 Additional methods

1.1 Efficient dimension reduction of the gene expression matrix

In the dimension reduction step, OCAT adopts the online Fast Similarity Matching (FSM) (Giovannucci et al., 2018) to efficiently project the normalized gene expression $X \in \mathbb{R}^M$ to its principal subspace $Y \in \mathbb{R}^d$. The online FSM algorithm is a fast and memory-efficient method for principal subspace projection (PSP), which outputs an updated estimate of the principal subspace after intaking a new datum (one cell at a time). The online FSM is an improvement over the Similar Matching (SM) (Pehleva et al., 2015) algorithm that solves

$$\min_{W} \max_{M} \ 2\mathrm{Tr}(W^T W) - \mathrm{Tr}(M^T M) - 2\sum_{t=1}^{N} \boldsymbol{x}_t^T W^T \boldsymbol{y}_t, \tag{1}$$

where $\boldsymbol{y}_t \equiv M^{-1} W \boldsymbol{x}_t$, for $t = 1, 2, \ldots, N$. As outlined in Algorithm 1, SM costs $O(Md + d^3)$ per iteration to solve

$$M\boldsymbol{y}_t = W\boldsymbol{x}_t. \tag{2}$$

When the reduced dimension $d \sim \sqrt{M}$, the cost $O(d^3)$ for solving is non-negligible. To accelerate the SM algorithm, the online FSM algorithm (Algorithm 2) adopts the Sherman–Morrison formula when updating M_{inv}, thus reducing the computing cost to $O(Md)$ per iteration.

Algorithm 1 Similarity Matching (SM) (Pehleva et al., 2015; Pehleva et al., 2017)

Input: Initial weights $M \in \mathbb{R}^{K \times K}$ and $W \in \mathbb{R}^{K \times D}$

for $t = 1, 2, 3, \ldots$ **do**
  $\boldsymbol{y}_t \leftarrow M^{-1} W \boldsymbol{x}_t$
  $W \leftarrow (1 - \alpha_t) W + \alpha_t \boldsymbol{y}_t \boldsymbol{x}_t^T$
  $M \leftarrow (1 - \beta_t) M + \beta_t \boldsymbol{y}_t \boldsymbol{y}_t^T$
end for

Algorithm 2 Fast Similarity Matching (FSM) (Giovannucci et al., 2018)

Input: Initial weights $M_{\text{inv}} \in \mathbb{R}^{K \times K}$ and $W \in \mathbb{R}^{K \times D}$

for $t = 1, 2, 3, \ldots$ **do**
  $\boldsymbol{y}_t \leftarrow M_{\text{inv}}^{-1} W \boldsymbol{x}_t$
  $M_{\text{inv}} \leftarrow \frac{1}{1-\beta_t} M_{\text{inv}}$
  $\boldsymbol{z}_t \leftarrow M_{\text{inv}} \boldsymbol{y}_t$
  $M_{\text{inv}} \leftarrow M_{\text{inv}} - \frac{\beta_t}{1+\beta_t \boldsymbol{z}_t^T \boldsymbol{y}_t} \boldsymbol{z}_t \boldsymbol{z}_t^T$
end for

1.2 Sparse graph construction through latent anchor embedding

OCAT constructs a sparse bipartite graph that connects each single cell to the "ghost" cell set. To compute the edge weights, $\boldsymbol{z}_{\langle i \rangle}$, between cell i and its s closest "ghost" cells, OCAT adopts the Local Anchor Embedding (LAE) algorithm (Liu et al., 2010) to optimize

$$\min_{\boldsymbol{z}_i \in \mathbb{R}^s} \quad \frac{1}{2}||\boldsymbol{y}_i - U_{\langle i \rangle}\boldsymbol{z}_{\langle i \rangle}||^2, \text{ such that } \mathbf{1}^T\boldsymbol{z}_{\langle i \rangle} = 1 \text{ and } \boldsymbol{z}_{\langle i \rangle} \geq 0, \tag{3}$$

and $U_{\langle i \rangle} = \{\boldsymbol{u}_k\}_{k \in \langle i \rangle}$ are the features of the s neighbor ghost cells.

LAE applies the projected gradient method to solve (3), and uses the Nesterov's method (Nesterov et al., 2003) to accelerate the gradient decent step (Algorithm 3). The LAE algorithm outputs a highly sparse weight matrix Z, with a memory usage of $O(sN)$ and time complexity $O(smN + s^2TN)$, where m is the total number of candidate "ghost" cells, s is the number of closest "ghost" cells to be selected, and T is the number of iterations. In practice, the LAE algorithm converges within a few iterations and T is therefore small. The LAE algorithm significantly accelerates the computational efficiency and reduces the memory usage of OCAT.

Algorithm 3 Local Anchor Embedding (LAE)

Input: data points $\{\boldsymbol{x}_i\}_{i=1}^n \subset \mathbb{R}^d$, anchor point matrix $U \in \mathbb{R}^{d \times m}$, integer s.

for i **to** n **do**
for $\boldsymbol{x}_i$ find s nearest neighbors in U, saving the index set $\langle i \rangle$;
define functions $g(z) = ||\boldsymbol{x}_i - U_{\langle i \rangle}\boldsymbol{z}||^2/2$, $\nabla g(\boldsymbol{z}) = U_{\langle i \rangle}^T U_{\langle i \rangle}\boldsymbol{z} - U_{\langle i \rangle}^T \boldsymbol{x}_i$, and $\tilde{g}_{\beta,\boldsymbol{v}}(\boldsymbol{z}) = g(\boldsymbol{v}) + \nabla g(\boldsymbol{v})^T(\boldsymbol{z} - \boldsymbol{v}) + \beta||\boldsymbol{z} - \boldsymbol{v}||^2/2$;
initialize $\boldsymbol{z}^{(0)} = \boldsymbol{z}^{(1)} = \mathbf{1}/s$, $\delta_{-1} = 0$, $\delta_0 = 1$, $\beta_0 = 1$, $t = 0$;
repeat
$t = t + 1$, $\alpha_t = \frac{\delta_{t-2} - 1}{\delta_{t-1}}$
set $\boldsymbol{v}^{(t)} = \boldsymbol{z}^{(t)} + \alpha_t(\boldsymbol{z}^{(t)} - \boldsymbol{z}^{(t-1)})$
for $j = 0, 1, \ldots$ **do**
$\beta = 2^j \beta_{t-1}$, $\boldsymbol{z} = \Pi_{\mathbb{S}}(\boldsymbol{v}^{(t)} - \frac{1}{\beta}\nabla g(\boldsymbol{v}^{(t)}))$
if $g(\boldsymbol{z}) \leq \tilde{g}_{\beta,\boldsymbol{v}^{(t)}}(\boldsymbol{z})$ **then**
update $\beta_t = \beta$ and $\boldsymbol{z}^{(t+1)} = \boldsymbol{z}$
break
end if
end for
update $\delta_t = \frac{1 + \sqrt{1 + 4\delta_{t-1}^2}}{2}$
until $\boldsymbol{z}^{(t)}$ **converges**;
$\boldsymbol{z}_i = \boldsymbol{z}^{(t)}$.
end for

Output: LAE vectors $\{\boldsymbol{z}_i\}_{i=1}^n$

2 Supplementary notes

2.1 Hyperparameter tuning

The OCAT package provides hyperparameter tuning functionalities for advanced users to examine multiple hyperparameter combinations and evaluate clustering performance based on suitable metrics.

To tune each hyperparameter m, d, or p, users may choose to input a list of values to test on. The OCAT tuning function returns suitable evaluation metrics to help users identify the best hyperparameter combinations. The OCAT tuning function evaluates hyperparameter setting through cell type clustering performance. If the annotated cell types are known, the tuning function returns NMI, AMI, and ARI scores as evaluation metrics compared with the known annotations. If the cell type annotations are unknown, the OCAT tuning function will estimate the number of clusters, and return the silhouette scores as evaluation metrics. A silhouette score close to 1 indicates that the clusters are distinct, while a silhouette score close to 0 means that the clusters are indifferent. Users can choose their ideal hyperparameter setting based on these evaluation metrics and their research goal.

Additionally, the OCAT tuning function can also recommend a range of values for the users to test on, as described in the Hyperparameter recommendations subsection in Methods and materials. For example, to identify the optimal hyperparameter settings of m, d and p for a dataset with 4,000 cells and 10,000 genes, the tuning algorithm will recommend the values of m from $\{25, 30, 35, 40, 45, 50\}$, d from $\{80, 90, 100, 110\}$ and p from $\{0.1, 0.3, 0.5\}$. The users may also fix any hyperparameter value if they do not need further tuning on it.
