# Supplementary material for: One Cell At a Time (OCAT): a unified framework to integrate and analyze single-cell RNA-seq data
Source: Genome Biol. 2022 Apr 20;23:102. doi: 10.1186/s13059-022-02659-1 (PMC9019955; doi:10.1186/s13059-022-02659-1)
Supplement: Supplementary file 3 — Additional file 3 Supplementary Figures S1-S10. [file 13059_2022_2659_MOESM3_ESM.pdf]

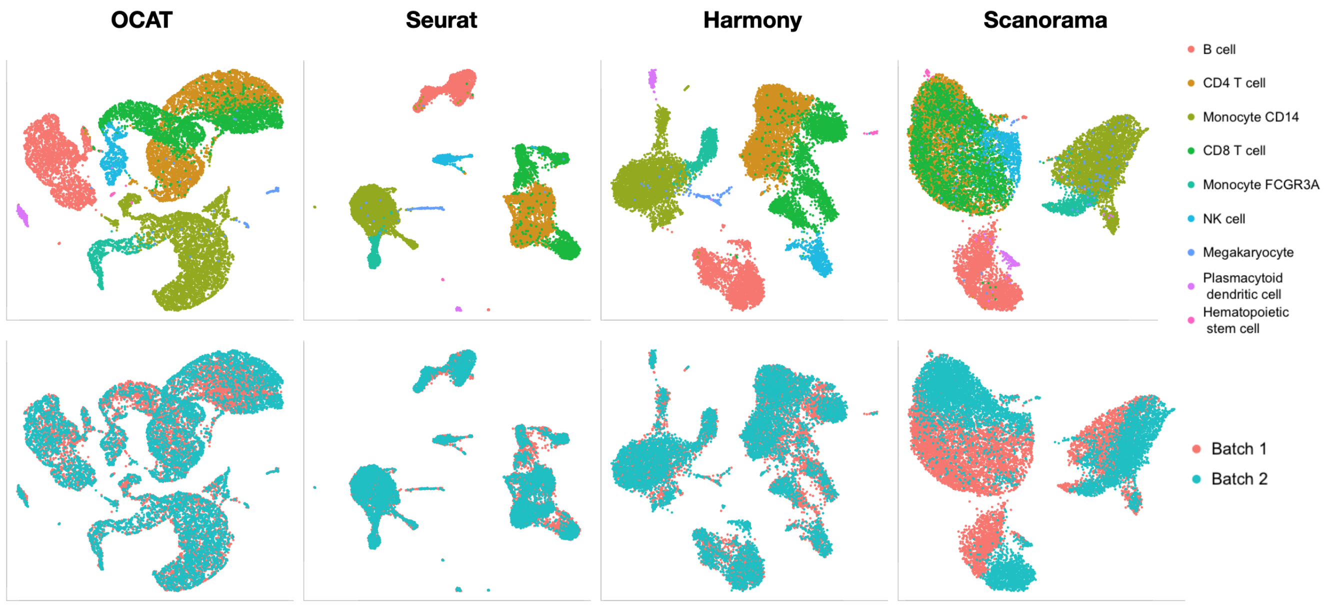

Fig S1: **UMAP projection of the integrated PBMC datasets using OCAT**, benchmarked with Seurat v3, Harmony and Scanorama. The top panels are colored by annotated cell types, and the bottom panels are colored by batch origins.

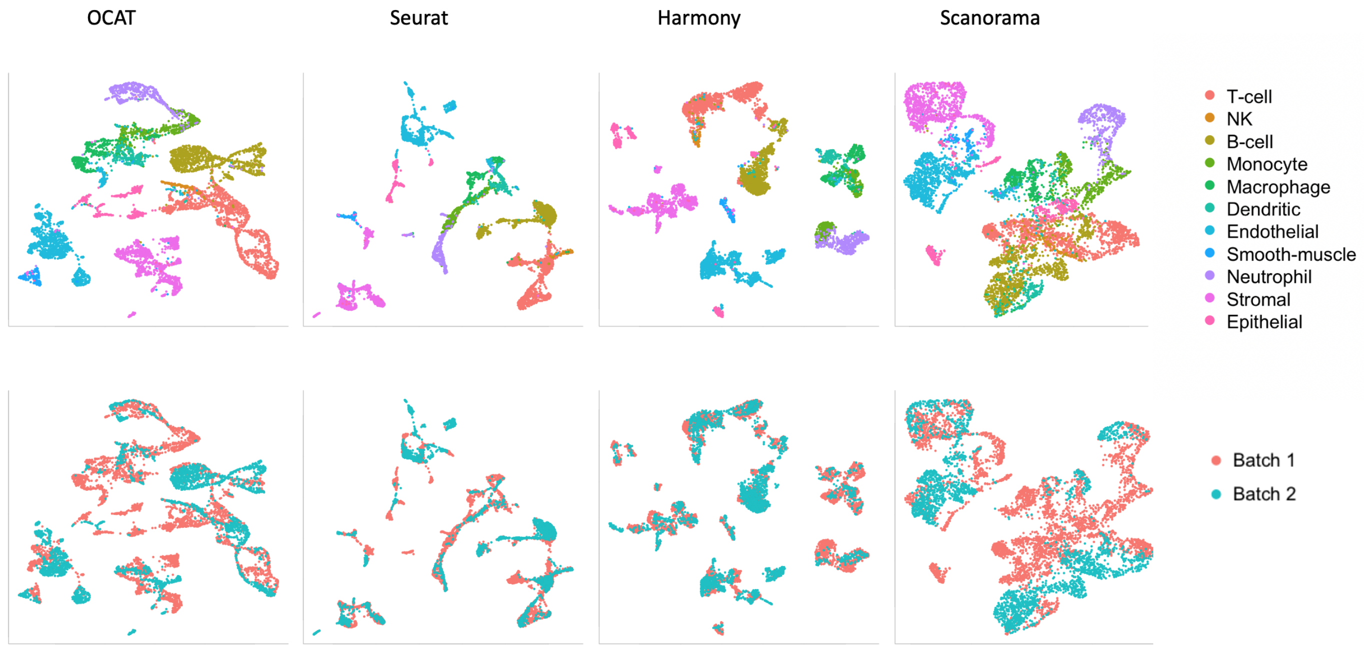

Fig S2: **UMAP projection of the integrated mouse atlas datasets using OCAT**, benchmarked with Seurat v3, Harmony and Scanorama. The top panels are colored by annotated cell types, and the bottom panels are colored by batch origins.

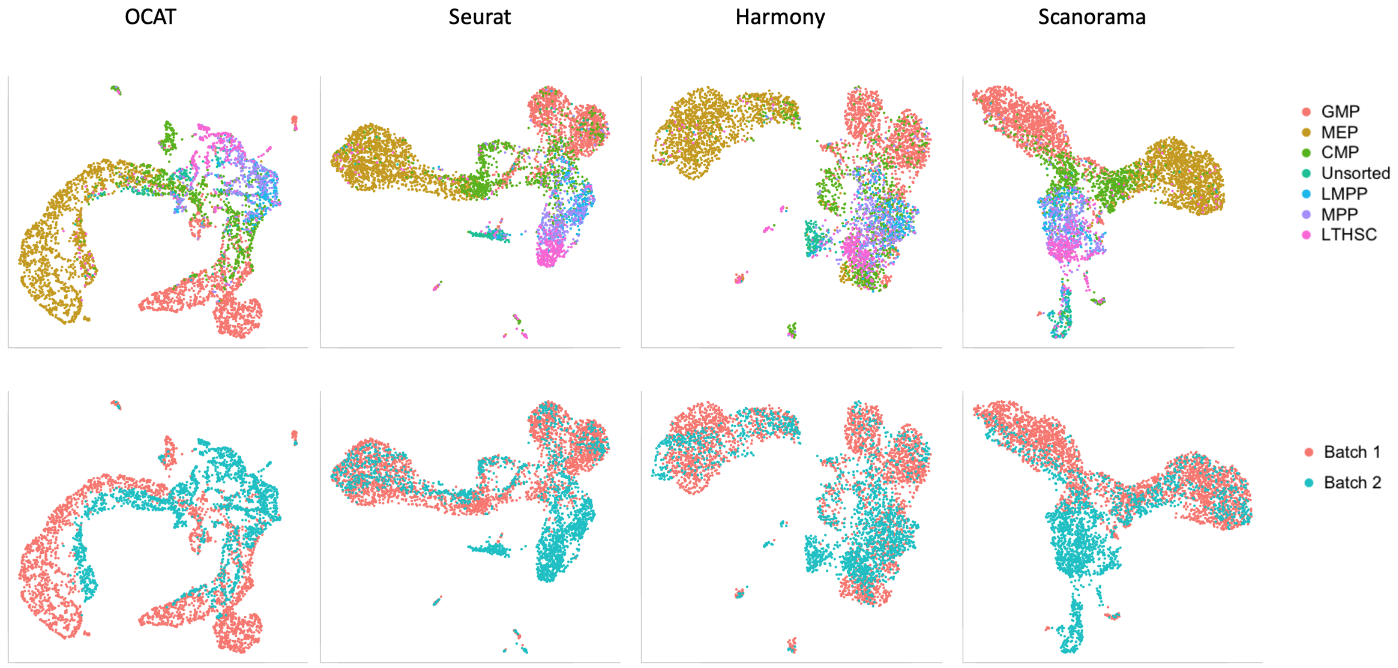

Fig S3: **UMAP projection of the integrated mouse hematopoietic datasets using OCAT**, benchmarked with Seurat v3, Harmony and Scanorama. The top panels are colored by annotated cell types, and the bottom panels are colored by batch origins.

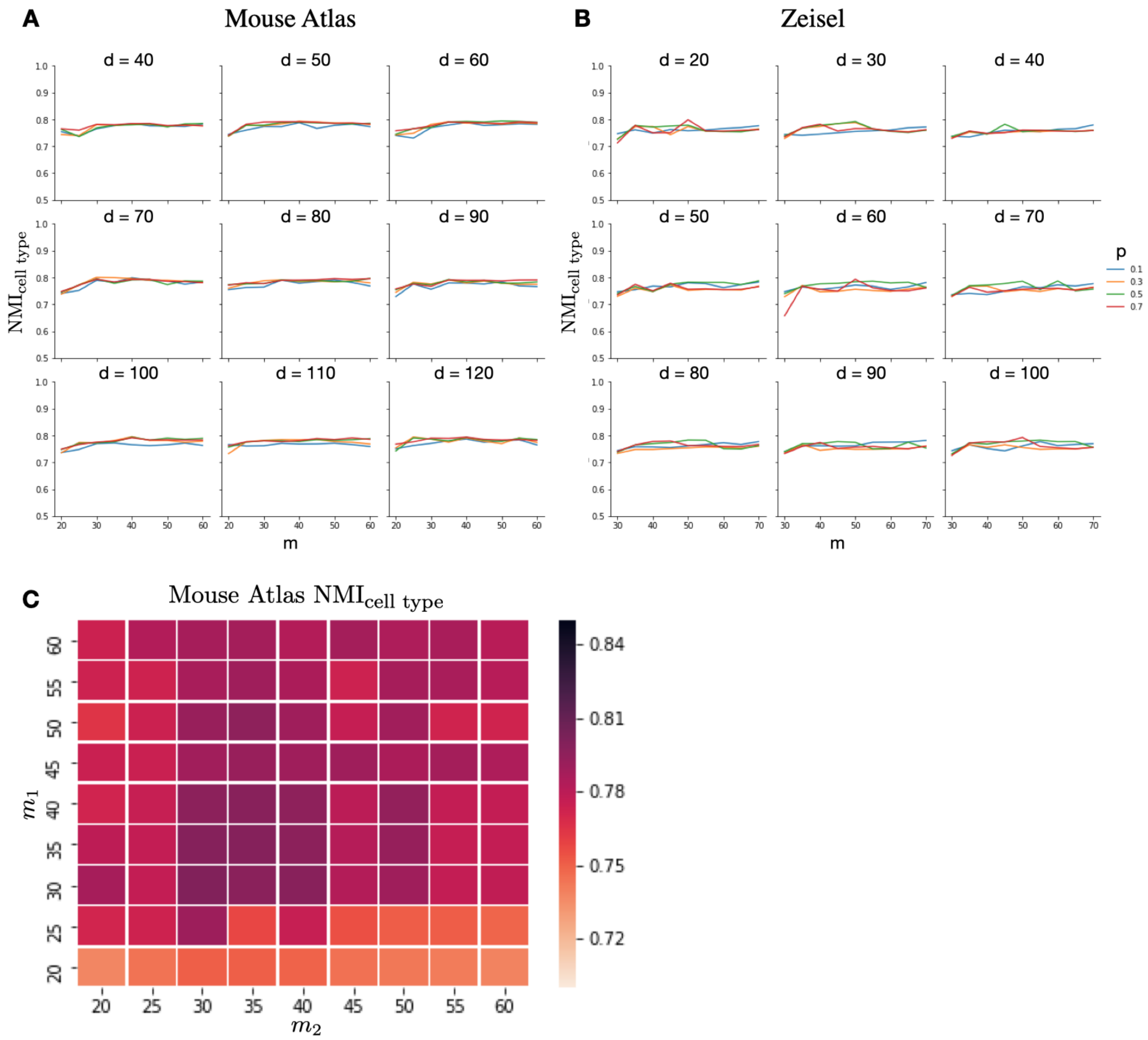

Fig S4: **OCAT hyperparameter sensitivity analysis.** **A**: Cell type clustering performance on integrating two mouse atlas datasets with different hyperparameter values of d, m, and p. The number of ghost cells m in both datasets are set identical. The panel reports $\mathrm{NMI}_{\text{cell type}}$ as the evaluation metric. **B**: Cell type clustering performance on the Zeisel dataset with different hyperparameter values of d, m, and p. The panel reports $\mathrm{NMI}_{\text{cell type}}$ as the evaluation metric. **C**: Heatmap of $\mathrm{NMI}_{\text{cell type}}$ on integrating two mouse atlas datasets with different choices for the number of "ghost" cells (i.e. m_1, m_2) for the two datasets. d is set to 70 and p is set to 0.3.

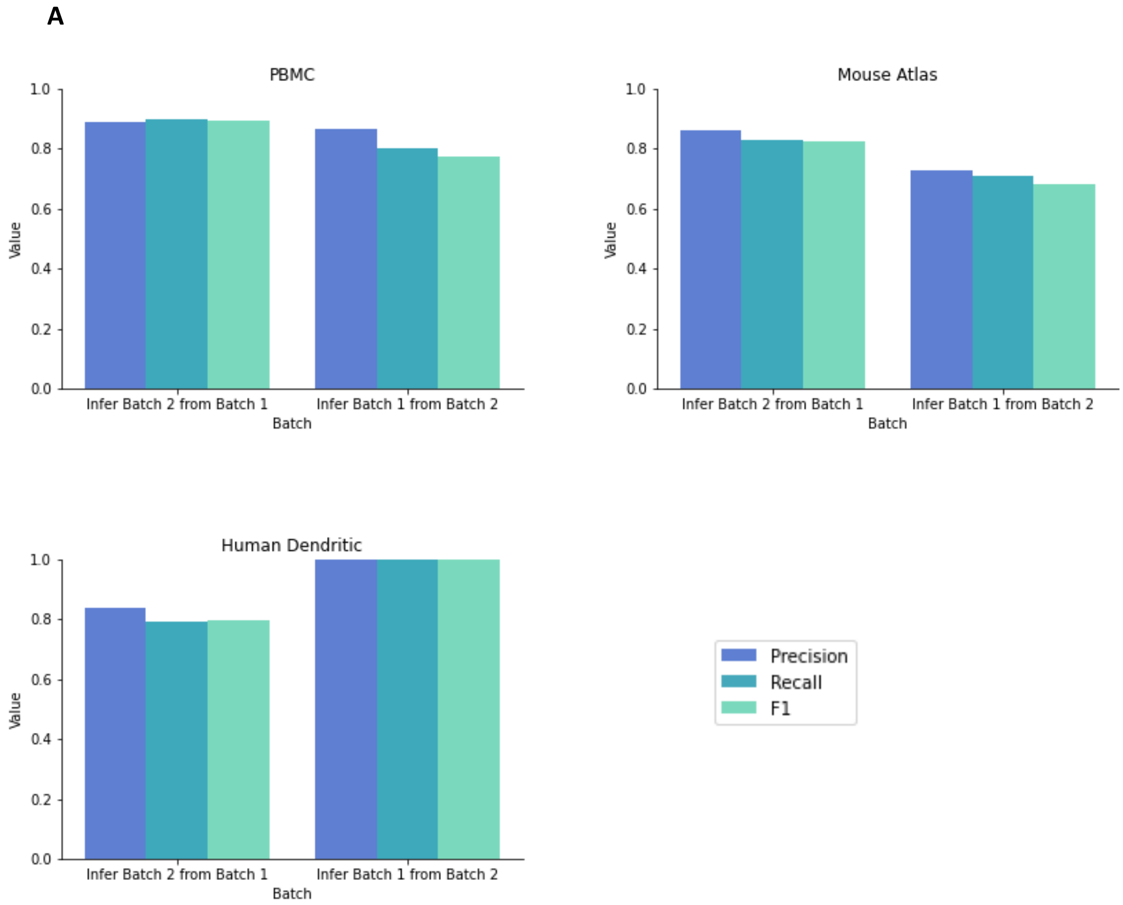

Fig S5: **OCAT cell type inference performance on integration datasets. A**: OCAT cell type inference performance on integration datasets. For each of the PBMC, mouse atlas and human dendritic datasets, we reported the Precision, Recall, and F1 scores on two sets of experiments (i.e. infer Batch 2 from Batch 1, infer Batch 1 from Batch 2).

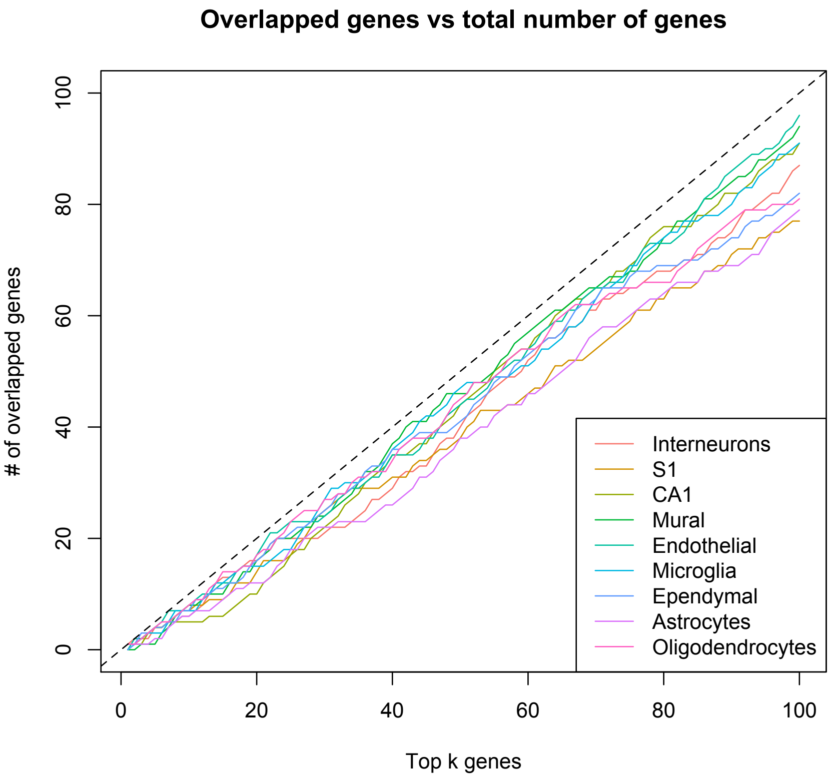

Fig S6: **Top differential gene overlap between OCAT and Seurat in the Zeisel dataset.** The number of top genes, k, ranges from 1 to 100. Each colored line represents one cell type group. The dotted diagonal line symbolizes perfect consistency between OCAT and Seurat. The closer a dot is to the diagonal line, the more overlaps between the top k differential genes selected by OCAT and Seurat v3. The top differential genes identified by OCAT and Seurat are mostly consistent for all cell types.

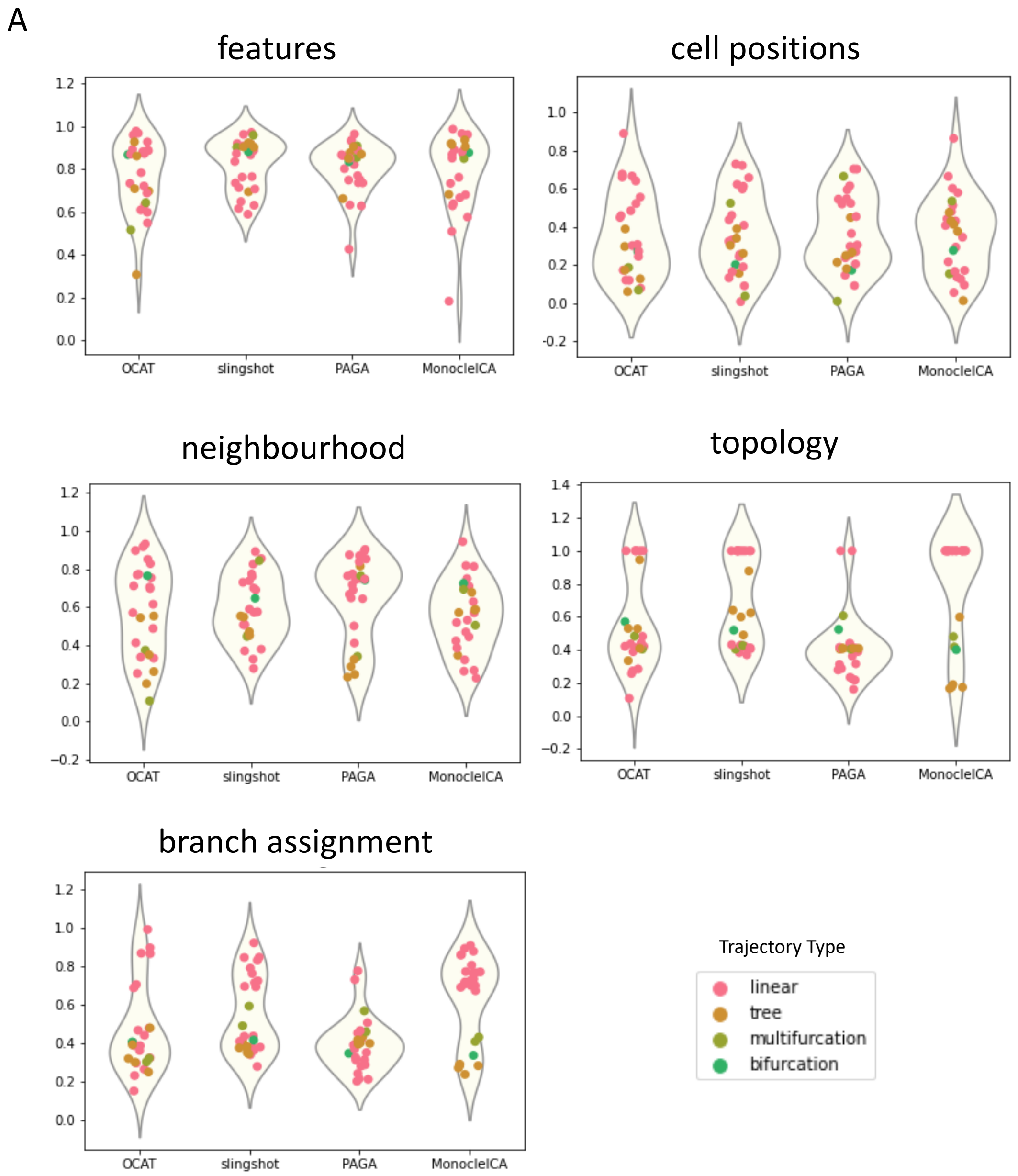

Fig S7: **OCAT trajectory and pseudotime inference benchmark on dynverse real datasets. A**: Violin plot of the OCAT trajectory and pseudotime inference metrics, benchmarking with Slingshot, PAGA and Monocole ICA. Each panel reports the distribution of each of the five aggregated metrics (features, cell positions, neighbourhood, topology, branch assignment) per dataset, color-coded by trajectory type (linear, tree, bifurcation, multifurcation).

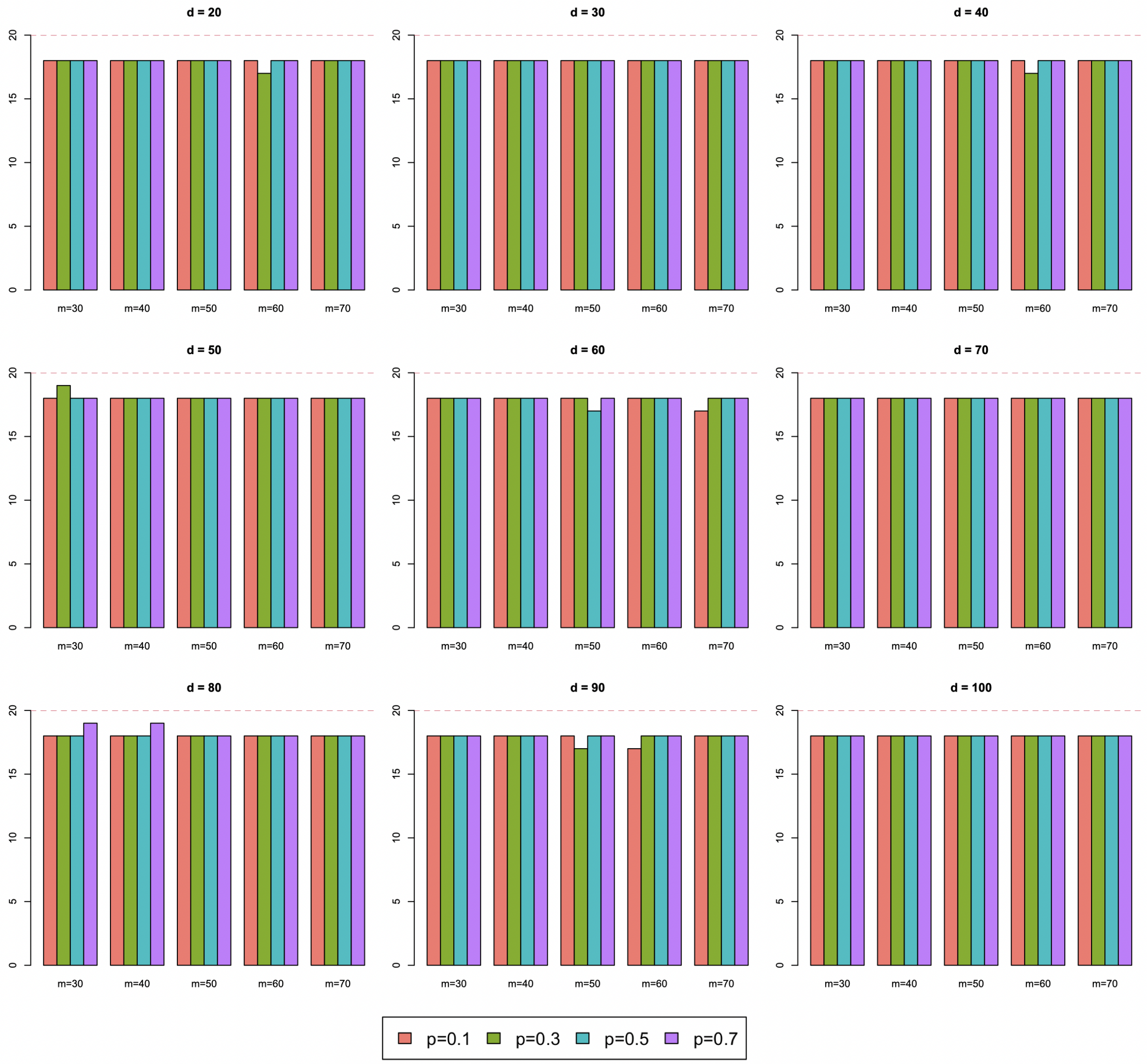

Fig S8: **OCAT sensitivity analysis of differential gene analysis using Zeisel dataset on the CA1 Pyramidal cell type.** Each bar represents the overlap in the top 20 differential genes using the corresponding hyperparameter setting with the top 20 genes identified using the original hyperparameter setting ($d = 30$, $m = 50$ and $p = 0.3$). The red horizontal dotted line symbolizes perfect consistency with the original hyperparameter setting. The overlap ranges between 17 to 19, which illustrates that the OCAT differential gene analysis is robust to hyperparameter choices.

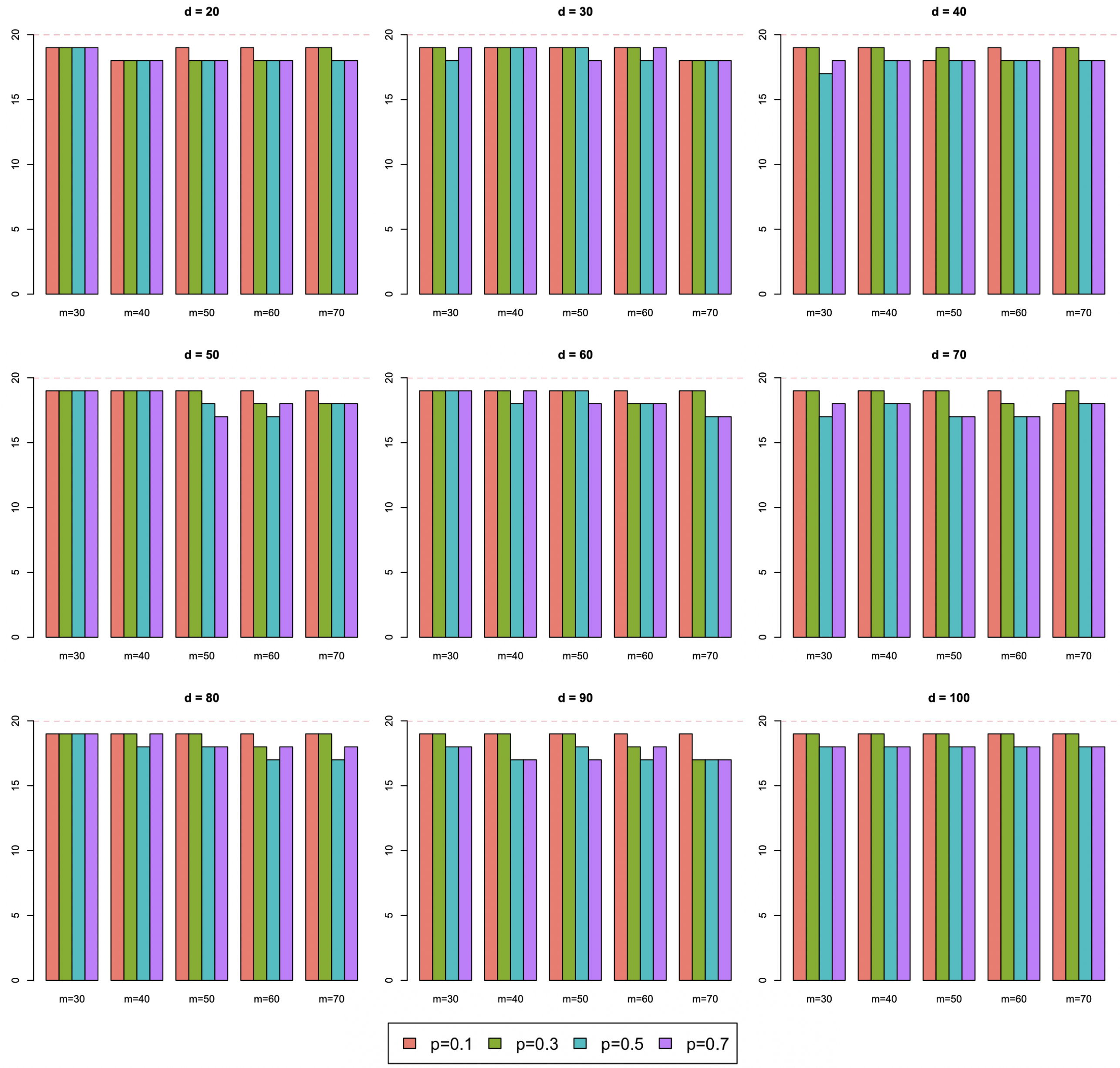

Fig S9: **OCAT sensitivity analysis of differential gene analysis using Zeisel dataset on the S1 Pyramidal cell type.** Each bar represents the overlap in the top 20 differential genes using the corresponding hyperparameter setting with the top 20 genes identified using the original hyperparameter setting ($d = 30$, $m = 50$ and $p = 0.3$). The red horizontal dotted line symbolizes perfect consistency with the original hyperparameter setting. The overlap ranges between 17 to 19, which illustrates that the OCAT differential gene analysis is robust to hyperparameter choices.

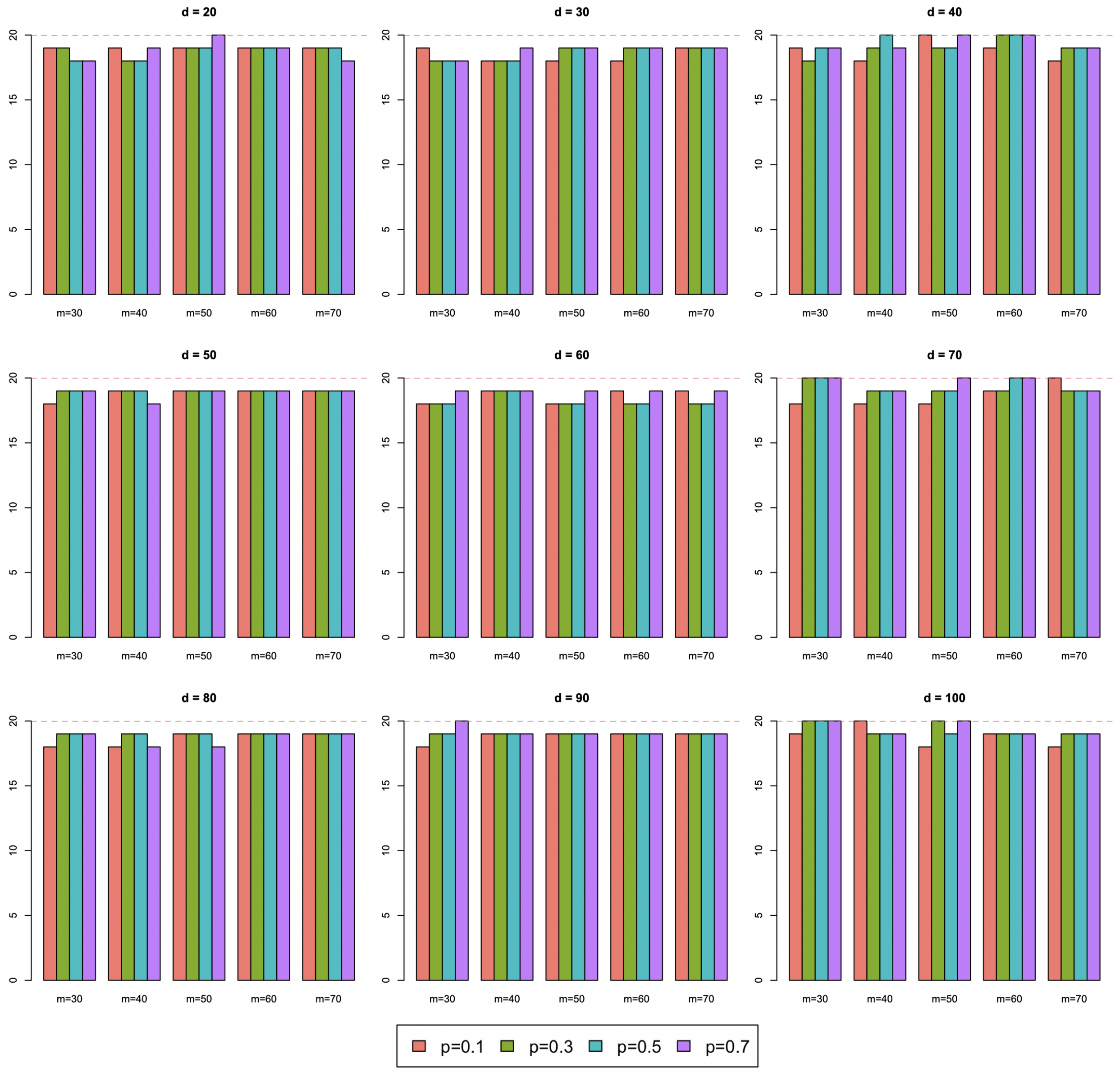

Fig S10: **OCAT sensitivity analysis of differential gene analysis using Zeisel dataset on the Interneurons cell type.** Each bar represents the overlap in the top 20 differential genes using the corresponding hyperparameter setting with the top 20 genes identified using the original hyperparameter setting ($d = 30$, $m = 50$ and $p = 0.3$). The red horizontal dotted line symbolizes perfect consistency with the original hyperparameter setting. The overlap ranges between 18 to 20, which illustrates that the OCAT differential gene analysis is robust to hyperparameter choices.
