# Supplementary material for: One Cell At a Time (OCAT): a unified framework to integrate and analyze single-cell RNA-seq data
Source: Genome Biol. 2022 Apr 20;23:102. doi: 10.1186/s13059-022-02659-1 (PMC9019955; doi:10.1186/s13059-022-02659-1)
Supplement: Supplementary file 4 — Additional file 4 Review history. [file 13059_2022_2659_MOESM4_ESM.docx]

Review History

**First round of review**

**Reviewer 1**

**Were you able to assess all statistics in the manuscript, including the appropriateness of statistical tests used?**

**Yes, and I have assessed the statistics in my report.**

**Comments to author:**

When integrating multiple scRNA-seq data sets, there are a few key challenges, such as missing values, feature selection and batch correction. Wang et al. proposed a new algorithm, One Cell At a Time (OCAT), to integrate and analyze scRNA-seq data sets to overcome some of the challenges. The algorithm has two outputs: the cell type-specific clusters and the spare encoding for downstream analysis tasks. From this sense, this is also a quite promising method for scRNA-seq data preprocessing. To compare the performance of the proposed method with other well-established methods, the authors made a comprehensive comparison analysis using multiple data datasets and demonstrated the advantages of proposed method. Overall, this is a well-designed and promising method for scRNA-seq data analysis, especially for integrating multiple scRNA-seq data sets.

I have below major comments.

Major issues

1. From Figure 1 - Schematic workflow of OCAT, the "ghost cells are data set-specific" (data sets 1 and 2 have 3 "ghost" cells, respectively, and the spare encoding includes 6 "ghost" cells). Based on the definition, the "ghost" cells are actually the data set-specific cluster centers or cell types. Hence, if we have two data sets from the same samples but generated by different technologies (or different batches as the manuscript mentioned), and each data set has 5 clusters (or "ghost" cells), the total number of clusters will be 10. Biologically speaking, since the two data sets are from the same samples, they should have some common or overlapped cell types (clusters or "ghost" cells). It is not clear how the OCAT can merge the same or similar cell types (clusters) across data sets when it integrates different data sets. For example, if we assume the above two data sets have 3 common cell types, then the number of cell types in the integrated data sets should be 7.

2. Still related to my question 1) discussed above, it looks the assignment of cells to clusters ("ghost" cells) is only decided in each individual data set using the k-means clustering approach. In the data integration stage (pages 11-12), the assignment of cells to clusters has not been updated. The only updated data is the spare embedding. Ideally, the assignment of the cells to the clusters should also be updated.

3. How the missing values are handled in the OCAT algorithm, especially when it applies to integrate multiple data sets.

4. Lines 12-13 of page 12 state "Following the message passing procedure outlined in Online Method Section 5.4.3, we obtain the refined embeddings, Zw^comb. This looks to be one of the core parts of the OCAT algorithm. It is necessary to detail the calculation of the refined embeddings in the method section. Furthermore, it is not sure whether the "Online Method Section 5.4.3" is a part of the current manuscript (did not find it in either main manuscript or Supplementary materials) or the original message passing paper (need to cite the paper here)?

Minor issues:

5. Some of the formulas have the equation numbers while others do not have. All of the formulas should be numbered.

**Reviewer 2**

**Were you able to assess all statistics in the manuscript, including the appropriateness of statistical tests used?**

**Yes, and I have assessed the statistics in my report.**

**Comments to author:**

The authors propose a new and computationally efficient approach to integrate multiple scRNA-seq data sets, as well as a framework for data analysis, ranging from cell type clustering, differential gene analysis, trajectory inference, pseudotime inference and cell inference. The method is benchmarked against existing methods in the field, and in multiple case studies. OCAT is based on sparse encoding and local anchor embedding to identify a set of ghost cells to reduce the dimension and complexity of the data. Importantly, the process avoids batch correction, which is often used in data integration methods, but may remove important biological variation.

The manuscript is clear and well written, and the examples are convincing. The proposed approach is original and covers a wide breadth of downstream analysis. The manuscript also considers a difficult data integration scenario when the datasets are not including the same cell types. My major comments are as follow.

1 - The cell type inference analysis needs to be extended. In the first scenario, datasets are split into 90% reference and 10% inference. Since each data set has been processed including all cells, that split already induces a positive bias for inference. I have a similar comment for the second scenario where PBMC datasets are considered, split into 90% 10% and 'cross-inferred'. A more realistic scenario would be to build a reference based on, say, two datasets, and an external data set as inference.

2 - The claim of 'potential to facilitate the discovery of de novo cell populations' is not substantiated. The downstream analyses rely on known cell types.

3 - The limitations of the approach are not acknowledged.

4 - I appreciated the hyper parameter sensitivity analysis. While the analysis shows that these parameters do not impact the NMI metrics (to assess predicted vs ground truth cell labels), the analysis should be extended for the some of the downstream analysis. For example, will the choice of d impact the identification of differentially expressed genes? There are no recommendations on how to choose the parameters, rather, an explanation of what that these parameters mean.

5 - It would be worthwhile to include a tuning function in the python package. While the manuscript shows examples where OCAT is robust against hyperparameter values, some advanced users may want to investigate different parameters for their own studies. Additionally, the m, d and s parameters could be added in Table 1 (currently in the supplemental table) to give some indications of which values might be suitable depending on data size.

6 - I understand that this paper is highly computational and methodological, however, some biological interpretation of the results is lacking to make it appealing to biologists.

7 - Currently, the section 'Dimension reduction of gene expression matrix' is quite unclear (that d is a chosen parameter d << M, and how y_i is defined), as the additional material is not referred.

Minor:

p5 line 18: [8] should be authors' names in full

p6 l57 efficaciously -> efficiently, scRAN-seq

p11 l13 as as -> as

p19, some values over the margin

p19 l56 the user selects

**Authors Response**

**Point-by-point responses to the reviewers’ comments:**

Reviewer #1:

When integrating multiple scRNA-seq data sets, there are a few key challenges, such as missing values, feature selection and batch correction. Wang et al. proposed a new algorithm, One Cell At a Time (OCAT), to integrate and analyze scRNA-seq data sets to overcome some of the challenges. The algorithm has two outputs: the cell type-specific clusters and the spare encoding for downstream analysis tasks. From this sense, this is also a quite promising method for scRNA-seq data preprocessing. To compare the performance of the proposed method with other well-established methods, the authors made a comprehensive comparison analysis using multiple data datasets and demonstrated the advantages of proposed method. Overall, this is a well-designed and promising method for scRNA-seq data analysis, especially for integrating multiple scRNA-seq data sets.

I have below major comments.

Major issues

1. From Figure 1 - Schematic workflow of OCAT, the "ghost cells are data set-specific" (data sets 1 and 2 have 3 "ghost" cells, respectively, and the spare encoding includes 6 "ghost" cells). Based on the definition, the "ghost" cells are actually the data set-specific cluster centers or cell types. Hence, if we have two data sets from the same samples but generated by different technologies (or different batches as the manuscript mentioned), and each data set has 5 clusters (or "ghost" cells), the total number of clusters will be 10. Biologically speaking, since the two data sets are from the same samples, they should have some common or overlapped cell types (clusters or "ghost" cells). It is not clear how the OCAT can merge the same or similar cell types (clusters) across data sets when it integrates different data sets. For example, if we assume the above two data sets have 3 common cell types, then the number of cell types in the integrated data sets should be 7.

*Response: We thank the reviewer for this comment. In OCAT, “ghost” cells are hypothetical cells that represent centers of small cell neighborhoods. These small cell neighborhoods may not necessarily be the cell type clusters. The number of “ghost” cells is a user-specific hyperparameter that may assume different values for the same dataset. For example, the number of “ghost” cells for the Zeisel dataset is 50, and it contains 9 annotated cell types. In Supplementary Figure S4B, we further showed that though we range the number of “ghost” cells from 30 to 70 for the Zeisel dataset, OCAT’s clustering performance remains consistent.*

*OCAT computes each cell’s connectivity to all the “ghost” cells, and uses the edge weights as the embedding of these cells. Each cell not only connects to the “ghost” cells from its own dataset, but also to those from other datasets to be integrated. These embeddings thus effectively capture cellto-cell similarity through this common set of “ghost” cells.*

*Take Figure 1 as a toy example. OCAT first identifies “ghost” cells 1, 2 and 3 from Dataset I, and ghost cells 4, 5 and 6 from Dataset II. For the yellow circle cell from Dataset I, OCAT connects it to ghost cells 1 and 2 (from Dataset I) and “ghost” cells 4 and 6 (from Dataset II), and uses the edge weights as the sparsified embedding for this cell. Similarly, for the yellow square cell from Dataset II, OCAT connects it to “ghost” cells 4 and 6 (from Dataset II) and ghost cells 1 and 2 (from Dataset I). Because both cells have strong connectivities to the same “ghost” cells, the cells are considered similar in transcriptomics, and are clustered to the same cell type (the blue clusters).*

*Through the edges connecting each cell to their selected “ghost” cells, OCAT manages to create a sparsified low-dimensional representation of each cell’s transcriptomics, correct batch effect and accurately clustering the cells. OCAT’s sparsified embedding makes it computationally fast and memory efficient. We also updated Figure 1 in the manuscript to clarify the schematic flow of OCAT.*

2. Still related to my question 1) discussed above, it looks the assignment of cells to clusters ("ghost" cells) is only decided in each individual data set using the k-means clustering approach. In the data integration stage (pages 11-12), the assignment of cells to clusters has not been updated. The only updated data is the spare embedding. Ideally, the assignment of the cells to the clusters should also be updated.

*Response: We thank the reviewer for this comment. In the OCAT pipeline, we use K-means clustering twice in two separate steps: 1) to identify “ghost” cells and 2) to cluster single cells. In step 1, the goal of using K-means clustering is to identify “ghost” cells in small cell neighborhoods and their feature representations. No clustering labels or cell type labels are assigned to cells at step 1. In step 2, with the OCAT final sparse embeddings of each cell, we use K-means to cluster the single cells, and assign clustering labels to the cells. K-means clustering here can be replaced by any other clustering algorithm, such as Louvain and Leiden, which are offered as alternatives in the OCAT package. Hence, the assignment of cluster labels to cells is only performed once, at step 2, using the OCAT final sparse embedding obtained in Equation (10). In the revised manuscript, we further clarified the use of K-means clustering algorithm in the Discussion section.*

3. How the missing values are handled in the OCAT algorithm, especially when it applies to integrate multiple data sets.

*Response: We thank the reviewer for this comment. So far, OCAT assumes that, when integrating multiple datasets, there exist shared signature genes that are aligned across all the datasets, which is a common assumption for most existing integration methods. If the gene expression of some cells is missing at random in one dataset, OCAT can easily adopt existing imputation methods (for example, KNN imputation) to “fill in” the missing values. However, if some major genes are systematically missing in one dataset (batch) but not in others, imputation may not work because the imputed gene expressions may be perfectly confounded with batch effect. As the technology of single-cell RNA sequencing advances rapidly, we argue that the most important (signature) genes are unlikely to be missing. Hence, using the common genes shared across all the datasets should be sufficient to characterize the gene expression of cells. In the revised manuscript, we have included how to handle missing values in the Discussion section.*

4. Lines 12-13 of page 12 state "Following the message passing procedure outlined in Online Method Section 5.4.3, we obtain the refined embeddings, Zw^comb. This looks to be one of the core parts of the OCAT algorithm. It is necessary to detail the calculation of the refined embeddings in the method section. Furthermore, it is not sure whether the "Online Method Section 5.4.3" is a part of the current manuscript (did not find it in either main manuscript or Supplementary materials) or the original message passing paper (need to cite the paper here)?

*Response: We thank the reviewer for this helpful comment. We have revised the Methods and materials section to describe the complete OCAT pipeline for integrating multiple scRNA-seq datasets. We also updated the Message passing between single cells subsection, and corrected the cross-reference of “Online Method Section 5.4.3”. The OCAT pipeline for analyzing individual scRNA-seq datasets can be considered as a special case of integrating multiple datasets by setting the number of datasets H = 1. We also added the citation of the original message passing paper to the revised Message passing between single cells subsection.*

Minor issues:

5. Some of the formulas have the equation numbers while others do not have. All of the formulas should be numbered.

*Response: We thank the reviewer for this comment. We have numbered all the formulas in the revised manuscript.*

Reviewer #2:

The authors propose a new and computationally efficient approach to integrate multiple scRNA-seq data sets, as well as a framework for data analysis, ranging from cell type clustering, differential gene analysis, trajectory inference, pseudotime inference and cell inference. The method is benchmarked against existing methods in the field, and in multiple case studies. OCAT is based on sparse encoding and local anchor embedding to identify a set of ghost cells to reduce the dimension and complexity of the data. Importantly, the process avoids batch correction, which is often used in data integration methods, but may remove important biological variation.

The manuscript is clear and well written, and the examples are convincing. The proposed approach is original and covers a wide breadth of downstream analysis. The manuscript also considers a difficult data integration scenario when the datasets are not including the same cell types. My major comments are as follow.

1 - The cell type inference analysis needs to be extended. In the first scenario, datasets are split into 90% reference and 10% inference. Since each data set has been processed including all cells, that split already induces a positive bias for inference. I have a similar comment for the second scenario where PBMC datasets are considered, split into 90% 10% and 'cross-inferred'. A more realistic scenario would be to build a reference based on, say, two datasets, and an external data set as inference.

*Response: We thank the reviewer for this comment. We have performed additional experiments on the Pancreas datasets to demonstrate the efficacy of OCAT in the suggested scenario. The new cell inference results are summarized in Supplementary Table S9, and for review convenience reproduced below. We included these new experiments and results in the Cell inference subsection in the Results section.*

*Specifically, we used the three largest Pancreas datasets (Baron, Muraro, and Segerstolpe) as reference, and tested cell type inference performance on two inference datasets (Xin or Wang) respectively. Each inference dataset is generated in a different experiment than reference datasets, and the reference contains all the cell types in the inference. We first integrated the three reference datasets (Baron, Muraro, Segerstolpe) to obtain the OCAT features from the combined reference set (n=12,818). We then trained an SVM on the combined reference set. For the first inference dataset Xin (n=1,492), we extracted the OCAT features and then used the trained SVM to assign labels on the inference OCAT features. It gives highly competitive classification performance (Precision=0.9945, Recall=0.9752, F1=0.9847).*

*In the second inference dataset, we performed the same procedure to infer the cell types of the Wang dataset (n=457) based on the same reference set (Baron, Muraro, Segerstolpe). It is worth noting that the Wang dataset is known to be noisy and that basic normalization fails to correct the distribution deviation from other datasets [1]. We would like to highlight that even in this challenging scenario, the OCAT cell inference method achieved high cell type prediction performance, with Precision=0.9103, Recall=0.8796, and F1=0.8932.*

2 - The claim of 'potential to facilitate the discovery of de novo cell populations' is not substantiated. The downstream analyses rely on known cell types.

*Response: We thank the reviewer for this helpful comment. As the main focus of the OCAT manuscript is on methodology development, we only presented the evaluation of OCAT using scRNA-seq datasets with annotated cell types so that we can benchmark OCAT’s performance against other state-of-the-art integration methods. We have demonstrated that OCAT is capable of integrating multiple datasets that consist of unshared cell types, whereas other methods failed. Based on this result, we envision that OCAT can retain de novo cell types as separate clusters when integrating multiple heterogenous scRNA-seq datasets. Furthermore, a recent study [2] deployed OCAT to integrate multiple scRNA-seq data of healthy human kidney from heterogeneous sources. They showed that OCAT identified a distinct resident macrophage population in the healthy kidney unique to their in-house dataset, which was validated and supported by experimental and biological insights. Since this paper has not been peer-reviewed, we removed this statement on discovery of de novo cell type to avoid confusion to the readers.*

3 - The limitations of the approach are not acknowledged.

*Response: We thank the reviewer for this helpful comment. In the original OCAT manuscript, we stated the assumptions of OCAT whereas it applies. In the revised manuscript, we summarized all the assumptions and limitations of OCAT in the Discussion section, and we also summarize the limitations below for convenience to review.*

*Shared genes. The current OCAT framework requires that the genes are aligned and shared across all the datasets, which is a common assumption for most existing integration methods. If some genes are missing from some datasets, OCAT cannot utilize the unshared genes. We argue that, as the advance of scRNA-seq technology, most signature genes are unlikely to be missing, which indicates using the common genes shared across all the datasets should be sufficient to characterize the gene expression of cells.*

*Missing values. If the expression of some cells is missing at random in one dataset, OCAT can easily adapt existing imputation methods to “fill in'' the missing values. If some major genes are systematically missing in one dataset (batch) but not in others, imputation may not work because imputation may be perfectly confounded with batch effect.*

*Trajectory and pseudotime inference. For the trajectory inference analysis, OCAT infers lineages to connect cell type clusters, which assumes the cell state of individual cells within the same cell type cluster is similar. OCAT also assumes that only one root cluster (least differentiated cluster) exists and produces more accurate prediction if the root cluster is known. For the pseudotime inference, OCAT assumes the pseudotime is linear with the degree of differentiation, but the true biological clock may not be so. It has been pointed out that pseudotime sometimes may not necessarily reflect the true biological differentiation timeline. In experiments where the temporal data is unknown, the inferred pseudotime would thus be challenging to be validated.*

4 - I appreciated the hyper parameter sensitivity analysis. While the analysis shows that these parameters do not impact the NMI metrics (to assess predicted vs ground truth cell labels), the analysis should be extended for the some of the downstream analysis. For example, will the choice of d impact the identification of differentially expressed genes? There are no recommendations on how to choose the parameters, rather, an explanation of what that these parameters mean.

*Response: We thank the reviewer for this comment. In the revised manuscript, we show that OCAT’s downstream analysis, such as differential gene expression analysis, is robust to hyperparameter selection. We have included this new sensitivity analysis in Hyperparameter sensitivity analysis in downstream tasks subsection in the Methods and materials section. We also included a new subsection Hyperparameter recommendations for users to select their desirable hyperparameters.*

*We conducted additional sensitivity analysis to demonstrate the robustness of OCAT to the choice of hyperparameters in downstream analysis, such as differential gene analysis. OCAT has three hyperparameters, d (the dimension of the reduced gene expression matrix), m (the number of ghost cells) and s (the number of most similar ghost cells). We also reparameterize s=p*m, where p represents the percentage of ghost cells selected to construct the sparsified embeddings. Users can fine-tune either p or s to adjust the sparsity of the OCAT embedding.*

*We used the Zeisel dataset as a demonstration. We first identified the top 20 most differentially expressed genes in the Zeisel dataset using OCAT with hyperparameters d=30, m=50 and p=0.3 (the original setting). We then identified the top 20 most differentially expressed genes for each cluster by taking the values d from {20, 30, 40, 50, 60, 70, 80, 90, 100}, the values of m from {30, 40, 50, 60, 70} and the values of p from {0.1, 0.3, 0.5, 0.7}, yielding a total of 180 parameter combinations. We compared the top 20 genes selected in each setting with the ones selected in the original analysis, and showed that the top 20 selected genes are highly consistent. We illustrated the number of overlapped genes in Supplementary Figures S8, S9 and S10 for three sample cell types: CA1 Pyramidal, S1 Pyramidal and Interneurons.*

*The choice of d should be based on the number of genes. In general, we recommend d to be around 1/100 times the number of genes, and the value of d should range from 30 to 150. The choice of m should be based on the number of cells in each dataset as well as the number of cell type clusters. We recommend m to be around 1/100 times the number of cells, and the value of m should range from 20 to 100. Another consideration when choosing m is the number of cell type clusters. We suggest increasing the value of m if the users desire more subclusters. For example, for a dataset with 5000 cells, we suggest m=50. However, if the users would like to see more than 30 subclusters, m should increase to 60 or 70. This general recommendation provides a guideline for users to navigate their exploratory analysis. For advanced users who wish to explore their hyperparameter settings, the OCAT package offers a hyperparameter tuning function; see Supplementary Notes 2.1 for details.*

5 - It would be worthwhile to include a tuning function in the python package. While the manuscript shows examples where OCAT is robust against hyperparameter values, some advanced users may want to investigate different parameters for their own studies. Additionally, the m, d and s parameters could be added in Table 1 (currently in the supplemental table) to give some indications of which values might be suitable depending on data size.

*Response: We thank the reviewer for this helpful comment. We have added the following tuning functionalities to OCAT: (i) tuning of m, the number of ghost cells; (ii) tuning of d, the dimension that the original gene expression is projected to; (iii) tuning of s, the number of selected ghost cells in the sparsified embeddings. In OCAT, we also reparameterize s=p*m, where p is the percentage of most similar ghost cells selected to construct the sparsified embeddings. Tuning p is equivalent to tuning s. OCAT by default fine-tunes p, and we thus describe the tuning procedures below using p instead of s. We have updated Table 1 with the hyperparameters used in each integration task. We also provide additional details on the hyperparameter tuning function in the Supplementary Notes 2.1 Hyperparameter tuning.*

*To tune each hyperparameter m, d, or p, users may choose to input a list of values to test on. The OCAT tuning function returns suitable evaluation metrics to help users identify the best hyperparameter combinations. The OCAT tuning function evaluates hyperparameter setting through cell type clustering performance. If the annotated cell types are known, the tuning function returns NMI, AMI, and ARI scores as evaluation metrics compared with the known annotations. If the cell type annotations are unknown, the OCAT tuning function will estimate the number of clusters, and return the silhouette scores as evaluation metrics. A silhouette score close to 1 indicates that the clusters are distinct, while a silhouette score close to 0 means that the clusters are indifferent. Users can choose their ideal hyperparameter setting based on these evaluation metrics and their research goal.*

*Additionally, the OCAT tuning function can also recommend a range of values for the users to test on, as described in the Hyperparameter recommendations subsection in Methods and materials. For example, to identify the optimal hyperparameter settings of m, d and p for a dataset with 4,000 cells and 10,000 genes, the tuning algorithm will recommend the values of m from {25, 30, 35, 40, 45, 50}, d from {80, 90, 100, 110} and p from {0.1, 0.3, 0.5}. The users may also fix any hyperparameter value if they do not need further tuning on it.*

6 - I understand that this paper is highly computational and methodological, however, some biological interpretation of the results is lacking to make it appealing to biologists.

*Response: We thank the reviewer for this helpful comment. We have added biological interpretations of the experiment results and guidelines for biologists to use OCAT for their own analyses. The updated material is included in the OCAT unifies various downstream biological inferences section. The biological new findings of McEvoy et al. [2] also support that OCAT has the great potential to facilitate biologists for novel discoveries using scRNA-seq datasets.*

7 - Currently, the section 'Dimension reduction of gene expression matrix' is quite unclear (that d is a chosen parameter d << M, and how y_i is defined), as the additional material is not referred.

*Response: We thank the reviewer for this helpful comment. We have updated the Dimension reduction of gene expression matrix subsection in Methods and materials section to clarify the dimension reduction step of OCAT, and use the notation 𝑥𝑖 ′ instead of 𝑦𝑖 in the updated manuscript to emphasize the connection between 𝑋 and 𝑋 ′ . We also added Supplementary Table S8 that summarizes the notations used in Methods and materials section.*

*Y is a low-dimension feature representation of the single cells. After pre-processing the raw scRNA-seq datasets, OCAT projects the original M-dimensional gene expression matrices to ddimensional representations of the cells. d is a user-specific hyperparameter, and we recommend d to be 1/100 times of M, the number of genes in the input gene expression matrices. 𝑦𝑖 (now renotated as 𝑥𝑖 ′ ) is obtained by optimizing*

*min 𝒙𝒊 ′∈ℝ 𝑑 ∑ Σ𝑖=1 𝑁 (𝒙𝑘 𝑻𝒙𝑘 − 𝒙𝑖 𝑻𝒙𝑖 ) 𝑁 𝟐 𝑘=1 , where N is the total number of cells.*

Minor:

p5 line 18: [8] should be authors' names in full

p6 l57 efficaciously -> efficiently, scRAN-seq

p11 l13 as as -> as

p19, some values over the margin

p19 l56 the user selects

*Response: We sincerely thank the referee for thoroughly reviewing our work and for pointing out the detailed typos and formatting issue. We hope the reviewer agrees that the revision is a muchimproved paper.*

*References 1. Pancreas - scRNA-seq Datasets. Available from: https://hemberglab.github.io/scRNA.seq.datasets/human/pancreas/#wang.*

*2. McEvoy, C.M., et al., Single-cell profiling of healthy human kidney reveals features of sex-based transcriptional programs and tissue-specific immunity. bioRxiv, 2021: p. 2021.12.09.471943.*

**Second round of review**

**Reviewer 1**

The authors have fully addressed my comments. I have no other further comments.

**Reviewer 2**

The authors have addressed my concerns. Congratulations on this great piece of work!
